# Non-targeted metabolomics and pseudo-targeted lipidomics combined with gut microbes reveal the protective effects of *Causonis japonica* (Thunb.) Raf. in ulcerative colitis mice

**DOI:** 10.3389/fcimb.2024.1397735

**Published:** 2024-10-15

**Authors:** Hua Huang, Jie Jiang, Yihua Fan, Xufeng Ding, Fang Li, Chuanxin Liu, Lijiang Ji

**Affiliations:** ^1^ Department of Anorectal Surgery, Changshu Hospital Affiliated to Nanjing University of Chinese Medicine, Changshu, Jiangsu, China; ^2^ Department of Rheumatism and Immunity, Hospital of Chengdu University of Traditional Chinese Medicine, Chengdu, Sichuan, China; ^3^ Medical Key Laboratory of Hereditary Rare Diseases of Henan, Luoyang Sub-Center of National Clinical Research Center for Metabolic Diseases, The First Affiliated Hospital, and College of Clinical Medicine of Henan University of Science and Technology, Luoyang, China

**Keywords:** ulcerative colitis, *Cayratia japonica* (Thunb.) Gagnep, metabolomics, lipidomics, gut microbiota

## Abstract

Ulcerative colitis (UC) is an inflammatory bowel disease characterized by recurrent inflammatory tissue damage to the intestinal mucosa and forming intestinal epithelial ulcers. It is one of the most intractable diseases in the world. To date, the mechanism is unclear. *Causonis japonica* (Thunb.) Raf. (Wu Lianmei in Chinese; WLM), a traditional Chinese medicine, which has a long history as an anti-inflammatory, but its effect on UC was unconfirmed yet. Therefore, we established a dextran sodium sulfate (DSS)-induced UC mice model and evaluated the therapeutic effect of WLM extract. The results indicated that WLM inhibits DSS-induced inflammatory response in colitis *in vivo*, decrease DSS-induced clinical manifestations, reverses colon length shortening, and reduces tissue damage. The results of ELISA kits suggested that WLM could reverse the levels of DSS-induced inflammatory factors. To explore the mechanism of WLM in treating DSS-induced UC, ^1^H NMR and UHPLC-Q/Orbitrap MS were used to perform non-targeted metabolomics analysis; 21 differential metabolites in colon tissues were closely related to UC. Meanwhile, the pseudo-targeted lipidomics based on UHPLC-Q/Trap MS was used to analyze lipid metabolism disorders, and 60 differential lipid compounds were screened. These differential compounds were mainly involved in glycerophospholipid, arachidonic acid, glycerolipid, citric acid, tyrosine, and ether lipid metabolisms. The analysis of gut microbial showed that WLM may improve the symptoms of UC mice by reducing the abundance of *Helicobacter* and *Streptococcus* and increasing the abundance of *Limosilactobacillus* and *Akkermansia*. Moreover, the real-time qPCR results showed that WLM extract could decrease the mRNA levels of inflammatory factors and may be associated with protecting the integrity of intestinal mucosal barrier by destroying *in vivo* metabolic pathways, especially by regulating energy and lipid metabolisms and reducing inflammatory reactions. It provides a beneficial reference for studying WLM to elucidate the therapeutic mechanism of UC.

## 1 Introduction

Ulcerative colitis (UC) is an idiopathic chronic relapsing-remitting inflammatory disease, with main clinical manifestations of abdominal pain, diarrhea, and mucinous bloody purulent stool. Its pathogenesis is accumulated in the rectal and colonic mucosa and submucosal layers (Liu Y. et al., 2022). The main etiology and pathogenesis of UC were unelucidated. UC could be caused by various factors such as environment, heredity, immunity, and infection; however, inflammation caused by an abnormal reaction of the intestinal mucosal immune system plays an essential role in the pathogenesis of UC (Ananthakrishnan, 2015; Dulai and Jairath, 2018; Safari et al., 2021). This global disease seriously affects the quality of life of patients and brings increasing troubles to human beings (Kaplan, 2015).

In herbal medicine, phenols, flavonoids, and alkaloids have excellent anti-inflammatory and anti-oxidant activity. They can regulate various inflammatory cytokines, such as tumor necrosis factor-α, interleukin-6, and cyclooxygenase-2 (COX-II). These excellent anti-inflammatories and anti-oxidant effects play a crucial role in the therapeutic process for UC (Zhang et al., 2016; Davatgaran-Taghipour et al., 2017). Wu Lianmei (WLM) is a plant of *cayratia* of the Vitaceae family and is used as root or whole grass, which has the effects of anti-inflammation (Gu et al., 1988), analgesic (Yan et al., 2013), and anti-bacterial (Deng et al., 2007). WLM is mainly composed of flavonoids, such as apigenin and genistein, which can effectively alleviate the clinical symptoms of UC by acting on the target pathways related to UC (Fu et al., 2022). However, the protective mechanism of WLM against inflammation in the colon remains largely unknown, especially in terms of inflammatory response and metabolism.

Metabolomics is a branch of system biology that provides new insights into disease biochemistry, toxicology, pharmacology, gene function, early diagnosis of diseases, and intestinal microorganisms by observing the changes in types and concentrations of metabolites (Alonso et al., 2015). Metabolomics aims to analyze the metabolites in biological samples qualitatively and quantitatively. However, due to the complex composition of biological samples, the comprehensive characterization of compounds is a great challenge (Filimoniuk et al., 2020). Mass spectrometer (MS) and nuclear magnetic resonance (NMR) were the main analytical platforms in metabolomics study, where NMR can be used for nondestructive analysis and annotation of metabolites; however, its detection sensitivity is relatively low (Wishart et al., 2022). Usually, mass spectrometry is combined with separation techniques, such as Gas chromatography-mass spectrometry (GC-MS) and liquid chromatogram-mass spectrometry (LC-MS), to analyze different target compounds (Sogin et al., 2019; Chen et al., 2022). LC-MS, compared to GC-MS, could analyze thermally unstable metabolites without derivatization and has the advantages of high throughput and high sensitivity. Therefore, LC-MS was the most commonly used tool in metabolomics research.

The intestinal tract contains a large number of microorganisms, which depend on the animal’s intestinal tract to live and help the host to fulfill a variety of physiological and biochemical functions. The intestine is not only an important digestive and absorptive place, but also the largest immune organ, which plays an extremely important role in maintaining the function of normal immune defense (Niu et al., 2023). As the largest and most complex micro-ecosystem in the human body, the gut microflora itself and its metabolites not only regulate human Health, but also play an essential role in bridging the gap between the diet and the host. Studies have shown that the imbalance of intestinal bacterial flora is related to the occurrence of ulcerative colitis, and the regulation of gut bacteria through drugs and beneficial bacteria can effectively alleviate the inflammation of the intestines (Cheng et al., 2022).

In our study, the anti-inflammatory effect of WLM was first evaluated. Meanwhile, the primary purpose of the study to screen the biomarkers in the colonic tissues of UC mice model treated with WLM based on a multi-platform (UHPLC-Q/Orbitrap MS and ^1^H-NMR) metabolomics approach and the pseudo-targeted lipidomics technology of UHPLC-Q/Trap MS. Furthermore, we explored the protective effect of WLM on DSS-induced UC mice from the perspective of the gut bacteria community. Additionally, we elaborated the mechanism of WLM on the therapeutic of UC from a perspective of molecular biology. These results provide a reference for the treatment of UC by WLM.

## 2 Materials and methods

### 2.1 Solvents and chemicals

High-performance liquid chromatography (HPLC) grade acetonitrile and methanol (Thermo Fisher, USA). Deuteroxide was purchased from Cambridge Isotope Laboratories, Inc (Tewksbury, USA). UNIQ-10 column Trizol total kit was provided by Sangon Biological Engineering Technology & Services Co., Ltd. (Shanghai, China). Mesalazine was purchased from Heilongjiang Tianhong Pharmaceutical Co., Ltd. The qualitative detection kit for fecal occult blood was provided by Beijing Reagan Biotechnology Co., Ltd. (Beijing, China). Dextran sodium sulfate was provided by Shanghai Yuanye Biotechnology Co., Ltd. (Shanghai, China).

### 2.2 Preparation of WLM aqueous extract

WLM for autumn harvest was purchased from Suzhou Tianling Traditional Chinese Medicine Co., Ltd (batch number: 200609). The voucher samples were stored in the sample room of Changshu Hospital of Traditional Chinese Medicine (CS-tcm20200023). An amount of 900 g of dried WLM whole stem was added with 9 L deionized water and extracted under reflux two times, 2 h each time, and the filtrate obtained from the two times was collected, concentrated, and dried. Finally, we obtained 100.2 g WLM water extract freeze-dried powder from 900g WLM, and the yield was 11.2%. In order to clarify the chemical composition of WLM extract, we used UHPLC-Q/Orbitrap mass spectrometry to analyze the composition of WLM extract. Chromatographic and Mass spectrometry condition parameters were presented in the **Supplementary Materials**.

### 2.3 Animal

The male C57BL/6J mice (20-22 g) were provided by Beijing HFK Bioscience Co., Ltd. (SCXK (Jing) 2019 - 0008). The mice were allowed to feed in standard feeding conditions. They were housed under a specific pathogen-free condition with a temperature of 25 ± 2°C, with alternating light/dark for 12 h, the humidity was about 50%–60%), and allowed a free diet and water. According to the Laboratory Animal Guide of National Institutes of Health, the experimental protocol was adopted by the Laboratory Animal Research by Changshu Hospital of TCM (2022042203).

### 2.4 Establishment and treatment of UC

The mice were randomly split into six groups (*n* = 10): normal control (Control), 3% DSS (Model), positive control (Mesalazine, 0.4 g/(kg·d)), WLM-low dosage (WLM-L, 0.35 g/(kg·d)), WLM-medium dosage (WLM-M, 0.7 g/(kg·d)), WLM-high dosage (WLM-H, 1.4 g/(kg·d)). The control was free to drink water; the remaining five groups were given a 3% DSS drinking solution for seven days, respectively. WLM and Mesalazine group mice were given intragastric treatment once a day for seven days. Meanwhile, the model and control group mice were administered the same amount of saline intragastric.

The mice were sacrificed at the end of experimental period, and the colon tissues, blood, and the cecum contents were collected. The cecum content was used to gut microbial analysis. The degree of colonic mucosal injury in each mouse was recorded and photographed. A part of the tissue (about 0.8 cm) was fixed with a 4% paraformaldehyde solution for pathological sections. The remaining colon segment was quickly frozen and stored at -80°C. To verify the safety of WLM, we used HE staining of heart, liver, spleen, lung, and kidney tissues of normal control C57 mice after continuous administration for seven days.

### 2.5 Evaluation of disease activity index in animal models

The body weight of mice was monitored daily during the experiment, and the general state of each mouse, such as hair, activity, hematochezia, and rectocele, was observed. The mice were scored according to the DAI standard (**Supplementary Table S1**). DAI index was measured for each mice every day according to the mice rate of weight loss, the degree of loose stools, and the degree of bleeding (Lv et al., 2021) as follows: DAI = (the mice rate of weight loss + the degree of loose stools + the degree of bleeding)/3.

### 2.6 Histological analysis

The collected colon tissues were fixed with 4% paraformaldehyde solution, embedded in paraffin, and sectioned for hematoxylin and eosin (H&E) staining. The microstructure of colon tissue was observed under a microscope. Moreover, the histopathological scores were measured through the scoring system referencing to previously reported (Jiang et al., 2021; Zhang CX. et al., 2022). The detail scoring system was shown in **Supplementary Material Table S2**.

### 2.7 ELISA analysis

Accurately, 50 mg of colon tissues were weighed and lysed in 9-fold amounts of RIPA buffer solution (Sorabio, Beijing, China). After incubation for 1 h at 4°C, the tissue sample was homogenized using an ultrasonic homogenizer for 60 s and centrifuged at 3000 rpm (5424R, Eppendorf) for 20 min at 4°C. Then, the contents of inflammatory cytokines (IL-2, TNF- α, IFN-γ, IL-1 β, IL-6, and IL-8) in the supernatant were evaluated using the ELISA kit.

### 2.8 Metabolomics analysis

#### 2.8.1 Metabolomic analysis based on ^1^H NMR

A 100 mg colon tissue was weighed and homogenized with ultrasound in 4-times ultra-pure water and centrifuged at 12000 rpm (5424R, Eppendorf) for 10 min at 4°C. A total of 400 μL acetonitrile was added into 100 μL supernatant to precipitate protein; all supernatants were blow-dried under dry nitrogen. Then, the blow-dried sample was dissolved in 500 μL Phosphate Buffered Saline (PBS; 0.15 mol/L K_2_HPO_4_-NaH_2_PO_4_, pH = 7.4, 10% D_2_O, 0.001% TSP), and 500 μL was aspirated and transferred for analysis.

^1^H NMR spectra of all samples were recorded through the Bruker AV600 spectrometer (Bruker Company, Germany) equipped with an ultra-low temperature probe. The colon tissue samples were acquired using a one-dimensional CPMGGPPR1D pulse sequence with presaturation during relaxation delay at 298 K. The cycle time (D1) and 90° pulse time (P1) were 1 s and 12.65 µs, respectively, the central excitation frequency (O1P) was 4.701, and the pre-saturated water peak suppression power (PLW9) was 35 dB. The number of sampling points was 64 k, the spectrum width (SW) was 12,019 Hz, and the number of scans (NS) was 128. Furthermore, J-RES, COSY, HMBC, and HSQC pulse sequences were performed for the signal annotation and identification of metabolites.

^1^H NMR data analysis was manual processing through MestReNova 9.0.1 software for phase and baseline corrections, normalization, and segment integration. Before normalization, the TSP and water signals in *δ* 0.00–0.50 and *δ* 4.55–5.0 were eliminated, respectively. Then, according to the method of total area normalization, segment integration was performed at intervals of 0.002 ppm. Multivariate statistical analysis was conducted with the SIMCA-P^+^ software (V 14.1, Umetrics, Sweden), including principal component analysis (PCA) and orthogonal partial least squares discriminant analysis (OPLS-DA). PCA is a multivariate statistical method to investigate the correlation between multiple variables, and the possible abnormal values were screened using the reduced-dimensional data analysis method. OPLS-DA, as a supervised analysis method, could screen the differences among groups. The biomarkers were selected based on the methods reported in the literature (Wang et al., 2020).

#### 2.8.2 UHPLC-Q/Orbitrap MS metabolomics

A total of 100 μL of colon tissue homogenate was mixed with 400 μL acetonitrile, which was swirled for 3 min and centrifuged at 14000 rpm (5424R, Eppendorf) for 10 min regarding previous methods (Wang et al., 2022). All supernatants were blow-dried under the flow of dry nitrogen. The dried samples were dissolved with 100 μL 80% methanol-water (methanol: water = 80: 20, *v/v*), vortexed for 5 min, and centrifuged at 14000 rpm (5424R, Eppendorf) for 10 min. Finally, 70 μL of the supernatants were taken for further analysis.

To accurately detect and stabilize the instrument, the quality control (QC) samples were prepared by mixing equal volumes of colon samples. The QC data were collected once every five samples during the sample detection period.

The LC data was collected through the UltiMate 3000 UHPLC system (Thermo Fisher Scientific) equipped with a HSS T_3_ column (2.1 × 100 mm, 1.8 μm) at 35°C. The injection volume was 2 μL. The mobile phase was 0.1% formic acid in water (A) and acetonitrile (B) with a flow rate of 0.4 mL/min. The gradient elution conditions were set as follows: 0 min, 3% B; 8 min, 100% B; 9 min, 100% B; 9.5 min, 3% B; 12 min, 3% B.

The MS analysis was performed on Q-Orbitrap MS (Thermo Fisher Scientific) using a HESI source, where the parameters were set as follows: source spray voltage was 3.5 and 3.0 kV in the positive and negative ions mode, respectively; the capillary temperature was 320°C, and aux gas heater temperature was 350°C, sheath gas (N_2_) was 35 L/h, and auxiliary gas (N_2_) was 10 L/h. The scanning mode was Full-ms/dd-ms^2^ acquired in both positive and negative ion modes simultaneously, and the detection range was *m/z* 100-1500. Data analysis and data acquisition were conducted through the Xcalibur 4.1 software.

The raw data of LC-MS was imported to the Compound Discover 3.1 software, which allowed alignment, peak selection, and filling gaps. All variables were normalized using the peak area sum normalization method. Then, the aligned data matrix was used for multivariate statistical analysis, and the same analysis method as the NMR method was adopted. The differential compounds were identified using the in-house database, retention time, accurate molecular weight, standard compound, and HMDB (https://hmdb.ca/) database.

#### 2.8.3 Lipidomics analysis

A total of 50 μL of colon tissue homogenate was mixed with 150 μL of cold isopropanol to extract the lipids metabolites (Tan et al., 2021). After being vortexed for 5 min, the homogenate was frozen at -20°C for 1 h and centrifuged at 14000 rpm (5424R, Eppendorf) for 20 min at 4°C. The supernatant was directly analyzed using an UHPLC-Q/Trap 6500^+^ MS. QC was collected by mixing equal amounts of colon homogenate.

Chromatographic separation was conducted using an LC-30AD system (SCIEX, Framingham, MA, USA) equipped with an ACQUITY UPLC BEH C_8_ column (Waters, 1.7 μm, 2.1 × 100 mm). The mobile phase was water-methanol-acetonitrile (3: 1: 1, *v/v/v*) with 5 mmol/L ammonium acetate (A) and isopropanol with 5 mmol/L ammonium acetate (B). The elution condition was as follows: colon samples 0–0.5 min, 20% B; 0.5–1.5 min, 20%–40% B; 1.5–3 min, 40%–60% B; 3–13 min, 60%–100% B; 100% B isocratic elution for 1 min; 20% B balanced for 3 min. The column temperature was adjusted to 40°C, the injection volume was 2.0 µL, and the flow rate was 0.30 mL/min.

The MS data were acquired using a triple quadrupole linear ion trap QTRAP 6500^+^ MS (SCIEX, USA). The spectral data acquisition was performed using a scheduled MRM with selected time windows in negative and positive ion modes. The MS parameters were set as follows: the gas temperature was 400 and 550°C, and the ion spray voltage was 5500 and 4500 V in positive and negative ion modes, respectively; GS1 and GS2, both 50 psi; CUR, 35 psi.

The lipidomics data were pretreated using Sciex OS software (V1.4, AB SCIEX, USA) by extracting the peak areas of target compounds by retention time. Data filled gap and normalization before the SIMCA-P+ software performed the statistical analysis. The pathway analysis was conducted through an online platform, MetaboAnalyst 5.0.

### 2.9 16S rRNA analysis

The total DNA from the cecum contents of mice was extracted using a DNA extraction kit. Subsequently, the V3-V4 region of the cecum contents was amplified by PCR after the extraction of total DNA. The PCR products were then used for library construction using the SMRT Bell method. The constructed libraries underwent initial quality inspection, and those passing the quality check were subjected to sequencing analysis using PacBio Sequel.

The raw data were processed using lima v1.7.0 software to identify Circular Consensus Sequencing (CCS) sequences. The CCS sequences were filtered using cutadapt 1.9.1 software, and UCHIME v4.2 software was employed to identify and remove chimeric sequences, resulting in operational taxonomic units (OTUs) suitable for subsequent analysis. Taking SILVA as a reference database, the naive Bayesian classifier combined with comparison method is used to annotate the feature sequence, and the species classification information corresponding to each feature can be obtained. The species abundance tables at different classification levels (phylum, class, order, family, genus, categories) can be generated by QIIME software. Finally, diversity analysis, differential analysis, correlation analysis, and functional prediction analysis were conducted.

### 2.10 Real-time PCR

To evaluate the mRNA expression, total RNA was extracted from colon tissue using the Trizol total RNA extraction kit (Sangon Biotech, China) (Zhao et al., 2021). The real-time RT-PCR was performed with Step One Plus thermocycler and PrimeScript RT Master Mix. **Supplementary Table S3** lists the sequences of primers. The mRNA expression of β -actin was used as an internal reference to normalize the target mRNA expression, and samples were analyzed using the 2^-ΔΔCT^ method.

### 2.11 Statistical analysis

All data were statistically analyzed using GraphPad Prism software v8.0 and expressed as mean ± SD. Statistically significant differences between groups were determined using a one-way analysis of variance (One-ANOVA). *P* < 0.05 was considered statistically significant.

## 3 Results

### 3.1 Chemical composition of WLM aqueous extract

The LC-MS methods established in this study were used to analyze WLM aqueous extract and standards. Combined with accurate relative molecular mass, fragment ions, and standard information from UHPLC-Q/Orbitrap-MS, a total of 25 chemical composition were identified (**Supplementary Table S4**). The total ion chromatograms (TIC) of WLM aqueous extract were demonstrated in **Supplementary Figure S1**.

### 3.2 WLM ameliorates symptoms of DSS-induced mice

**Figure 1:**
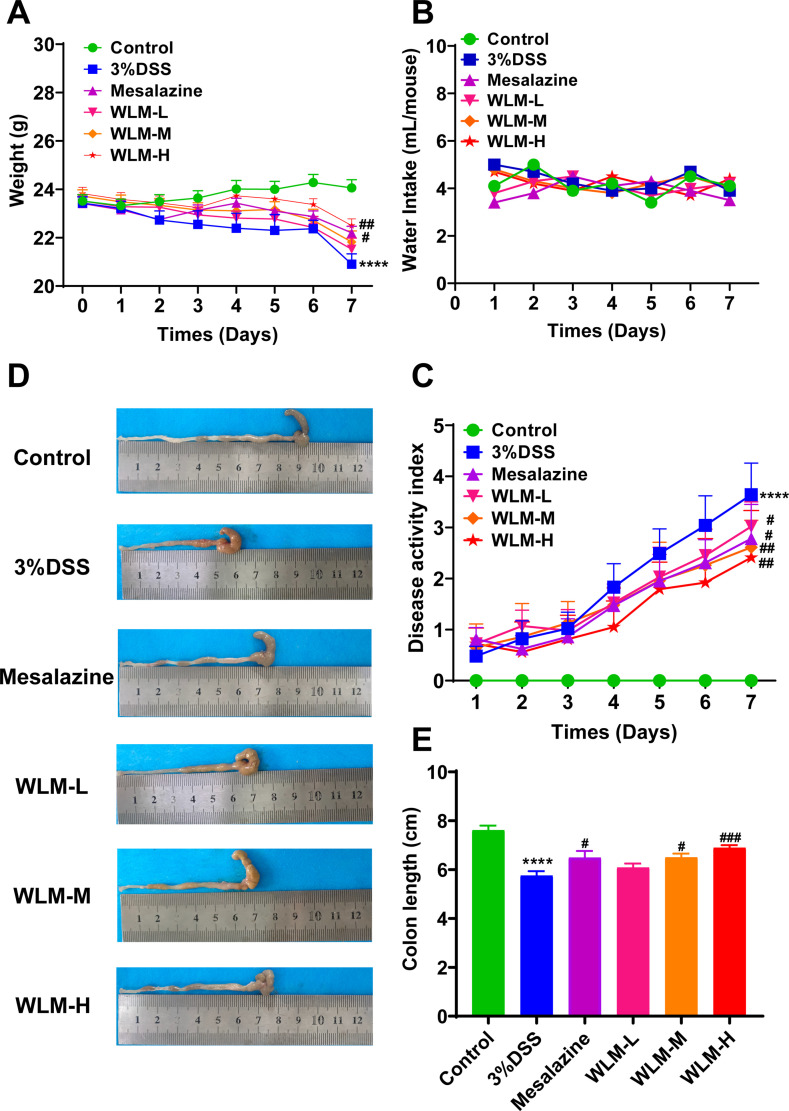
WLM ameliorates the symptoms of DSS-induced UC mice. **(A)** Body weight change; **(B)** water intake; **(C)** DAI; **(D, E)** colon length. # *p* < 0.05, ### *p* < 0.001 versus Control, **** *p* < 0.0001 versus Model by One-Way ANOVA followed with the Tukey’s *post-hoc* test.

To validate whether WLM could improve colonic damage in UC mice, we assessed the effect of WLM extracts through 3% DSS-induced mice. The results indicated that WLM significantly improved DSS-induced body DAI index and weight loss, but the water intake in mice was significantly unaffected (**Figures 1A–C**). Moreover, WLM treatment remarkably reversed the length shortening of the UC mice colon (**Figures 1D, E**), which was dose-dependent, and the treatment effect of high dose group was better.

### 3.3 WLM alleviates inflammatory damage in DSS-induced mice

**Figure 2:**
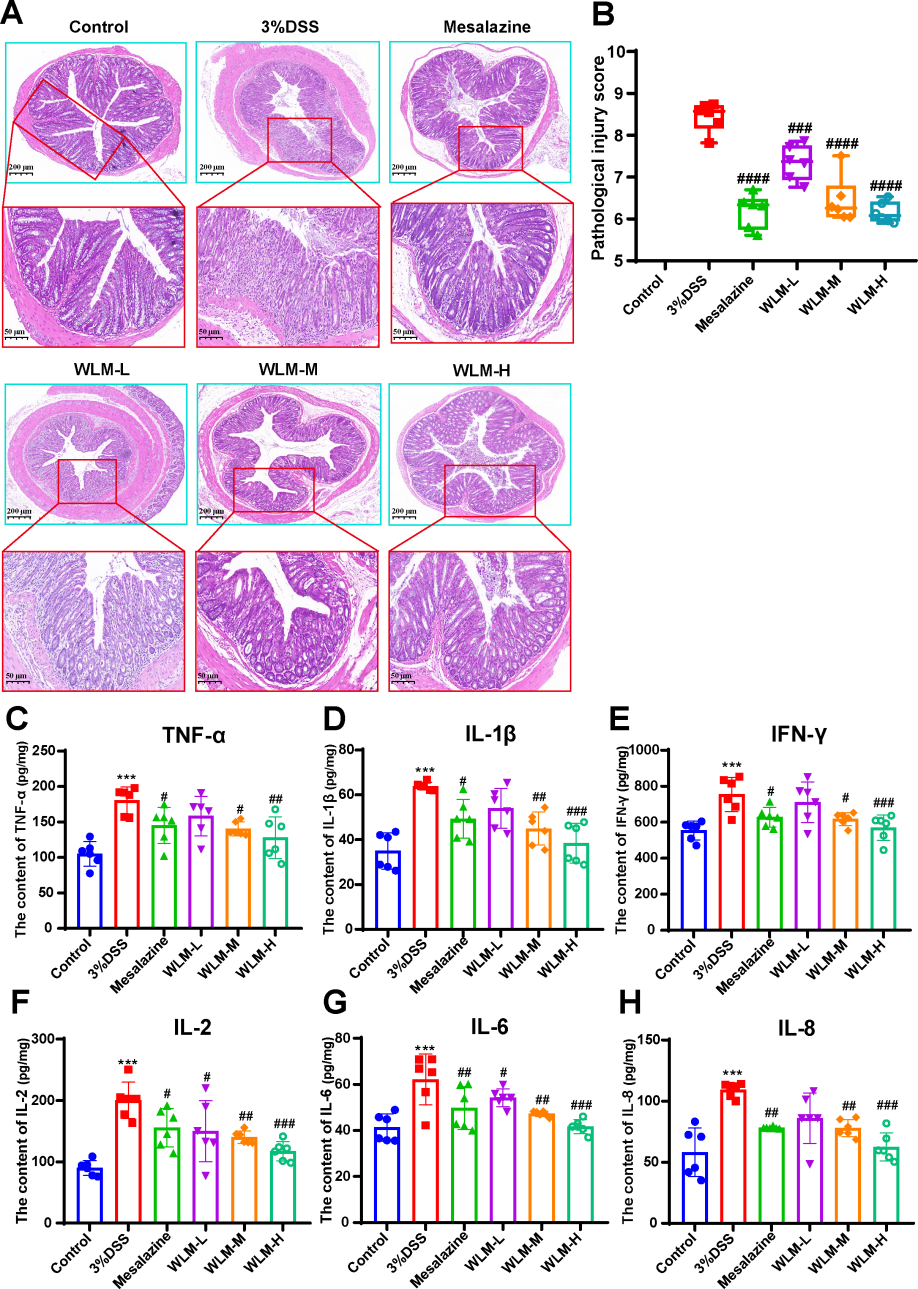
H&E-staining and WLM exhibited anti-inflammatory effects in DSS-induced UC mice. **(A, B)** H&E-staining and pathological injury score; **(C–H)** WLM suppressed pro-inflammatory cytokines, including TNF-alpha, IL-1β; IFN-γ; IL-2; IL-6; and IL-8 in colonic tissues of DSS-induced UC mice. # *p* < 0.05, ## *p* < 0.01, ### *p* < 0.001, #### *p* < 0.0001 versus Control, *** *p* < 0.001 versus Model by One-Way ANOVA followed with the Tukey’s *post-hoc* test.

Histopathological results indicated that the villous cells were denatured and necrotic compared to the Control group. Meanwhile, many inflammatory cells proliferated and infiltrated the colon tissue of UC mice (**Figures 2A, B**). However, the tissues of the Mesalazine, the WLM-M, and the WLM-H were normal physiologically after treatment, and no obvious pathological changes were observed in WLM-H group. To verify the safety of WLM, we used HE staining of heart, liver, spleen, lung, and kidney tissues of normal C57 mice after continuous administration for seven days. The results showed that there was no significant damage to each tissue, and it was safe and non-toxic at high dose (**Supplementary Figure S1**). Furthermore, the inflammatory factor levels of IL-2, IL-1β, IFN-γ, IL-6, TNF-α, and IL-8 in the colon were detected. The ELISA results showed that WLM extract decreased these inflammatory cytokines in a dose-dependent manner (**Figures 2C–H**), and the effect was more significant in the high-dose group. Therefore, the follow-up metabolomics, lipidomics and gut microbiota analysis were all analyzed in the high-dose group.

### 3.4 Metabolomic analysis of colon sample based on UHPLC/Q-Orbitrap MS

To explore the mechanism of WLM exact in the treatment of UC, we used WLM-H group to carry out the detects. A total of 3371 characteristic ion peaks were detected in the colon tissue sample through the UHPLC-MS. **Supplementary Figure S2** shows representative total ion chromatogram of colonic tissue in the WLM-H, model, and control groups.

**Figure 3:**
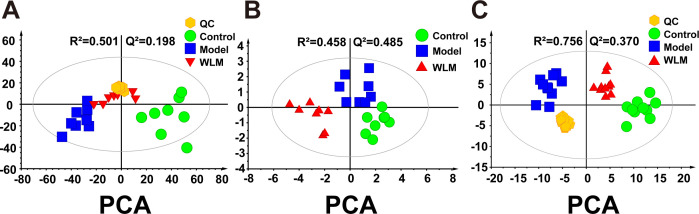
The principal component analysis of UC mouse colon tissue based on UHPLC-MS and ^1^H NMR. **(A)** PCA score plots of UHPLC-Q/Orbitrap MS metabolomics from colonic tissue of UC mice; **(B)** PCA score plots of ^1^H NMR metabolomics from colonic tissue of UC mice; **(C)** PCA score plots of UHPLC-Q/Trap MS lipidomics from colonic tissue of UC mice.

**Figure 4:**
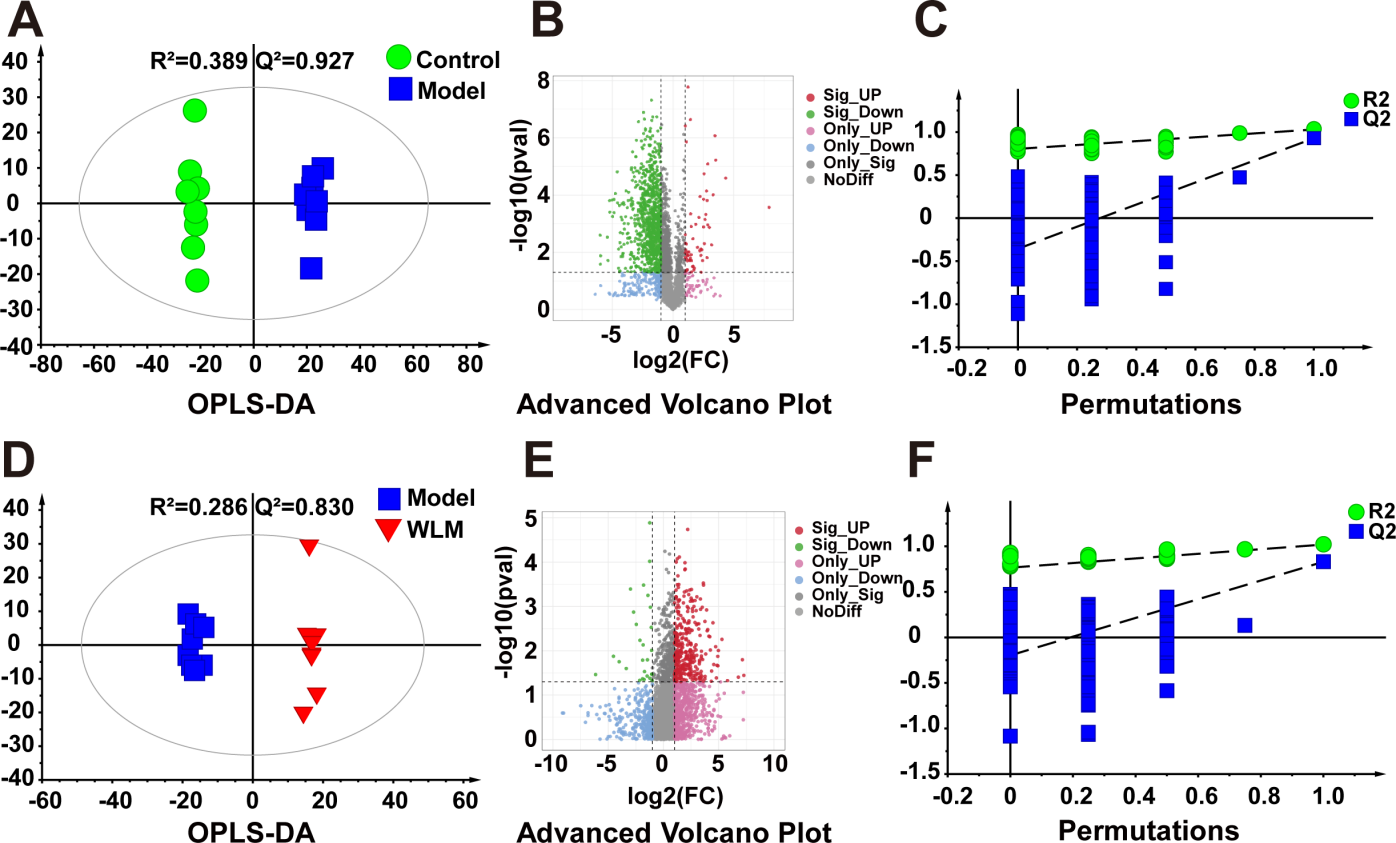
The OPLS-DA score plots. **(A, D)** Volcano plot **(B, E)** and permutations analysis **(C, F)** of the UHPLC-Q/Orbitrap MS spectra from colonic tissue of UC mice.

**Figure 3A** illustrates the multivariate statistical analysis PCA plot of the MS data. Control, 3% DSS, and WLM groups were separated, indicating significant differences in metabolites between groups in colonic tissue. To better screen the differential metabolites of UC, the MS database was further analyzed through the volcano plot (**Figures 4B, E**), and OPLS-DA (**Figures 4A, D**). Furthermore, the permutation analysis was used to verify if the OPLS-DA model was over-fitted, and the results showed that OPLS-DA showed no over-fitting (**Figures 4C, F**). Differential metabolites were selected based on VIP > 1.0, FC > 2.0, and *p*-values (*p* < 0.05). **Table 1** lists all significant differential metabolites from UHPLC/Q-Orbitrap MS.

**Table 1:** Based on UHPLC-Q/Orbitrap MS for differential metabolites of mice colon.

No.	Metabolites	Formula	MW.	RT (min)	DSS/Con	Log_2_ FC	p-value	WLM/DSS	Log_2_FC	p-value		HMDB
1	Cholic acid	C_24_ H_40_ O_5_	408.28700	5.70	0.19	-2.37	1.84E-04	8.38	3.07		2.58E-02	HMDB0000619
2	13-HpODE	C_18_H_32_O_4_	312.22973	6.08	0.22	-2.22	3.35E-04	5.39	2.43		9.41E-03	HMDB0062434
3	LysoPE(18:2/0:0)	C_23_H_44_NO_7_P	477.28518	6.68	0.43	-1.23	7.56E-03	3.15	1.66		1.06E-02	HMDB0011507
4	LysoPE(20:2/0:0)	C_25_H_48_NO_7_P	505.31639	6.71	0.35	-1.52	2.24E-03	4.28	2.10		4.47E-04	HMDB0011513
5	LysoPC(20:4)	C_28_H_51_NO_7_P	543.33201	6.75	0.25	-1.98	2.77E-03	2.73	1.45		4.14E-02	HMDB0010396
6	LysoPC(22:6)	C_30_H_51_NO_7_P	567.33201	6.75	0.28	-1.85	1.36E-05	2.55	1.35		7.69E-04	HMDB0010404
7	LysoPC(18:2)	C_26_H_51_NO_7_P	519.33212	6.76	0.25	-2.02	1.74E-03	3.37	1.75		5.03E-03	HMDB0010386
8	LysoPC(22:5)	C_30_H_53_NO_7_P	569.34799	6.95	0.30	-1.76	8.25E-05	6.43	2.68		1.71E-03	HMDB0010403
9	LysoPC(14:0)	C_22_H_47_NO_7_P	467.30069	7.22	0.45	-1.14	2.49E-03	7.24	2.86		7.34E-04	HMDB0010379
10	LysoPC(22:4)	C_30_H_55_NO_7_P	571.36326	7.37	0.32	-1.63	3.80E-05	2.25	1.17		1.11E-02	HMDB0010401
11	LysoPC(16:1)	C_24_H_49_NO_7_P	493.31640	7.41	0.36	-1.45	8.89E-03	6.23	2.64		7.56E-04	HMDB0010383
12	LysoPE(22:4/0:0)	C_27_H_48_NO_7_P	529.31640	7.42	0.27	-1.91	1.46E-04	2.94	1.56		1.35E-02	HMDB0011523
13	Docosahexaenoyl Ethanolamide	C_24_H_37_NO_2_	371.28215	8.01	0.13	-2.93	4.98E-04	4.42	2.14		1.25E-02	HMDB0013658
14	15(S)-HETE	C_20_H_32_O_3_	320.23484	8.43	0.08	-3.73	3.24E-03	4.47	2.16		1.01E-03	HMDB0003876
15	LysoPC(18:1/0:0)	C_26_H_53_NO_7_P	521.34774	7.24	0.35	-1.52	9.66E-05	2.50	1.32		1.22E-02	HMDB0002815
16	13(S)-HpOTrE	C_18_H_30_O_4_	310.21402	8.98	0.12	-3.01	1.44E-05	26.45	4.73		8.07E-03	HMDB0301803

Dss, DSS group; Con, Control group; WLM, WLM high dose group; FC, Fold Change.

A total of 16 differential metabolite contents were significantly disturbed. Compared to the Control group, the downregulated levels of 13-HpODE, 13(S)-HpOTrE, 15(S)-HETE, Cholic acid, docosahexaenoyl ethanolamide (DHEA), adrenic acid, LysoPC(14:0), LysoPE(22:0/0:0), LysoPE(22:4/0:0), LysoPC(16:1), LysoPC(18:2), LysoPC(22:4), LysoPC(22:5), LysoPC(22:6), LysoPE(18:2/0:0), LysoPC(20:4), LysoPE(20:2/0:0), LysoPC(18:1/0:0), norepinephrine, palmitoylcarnitine, uridine. However, the levels of these differential metabolites were significantly elevated after treatment with WLM extracts. **Supplementary Figure S3** depicts the relative content levels of 16 potential biomarkers.

### 3.5 Metabolomic and lipidomic profiling

#### 3.5.1 Metabolomic alteration of colon tissues based on ^1^H NMR

**Figure 5:**
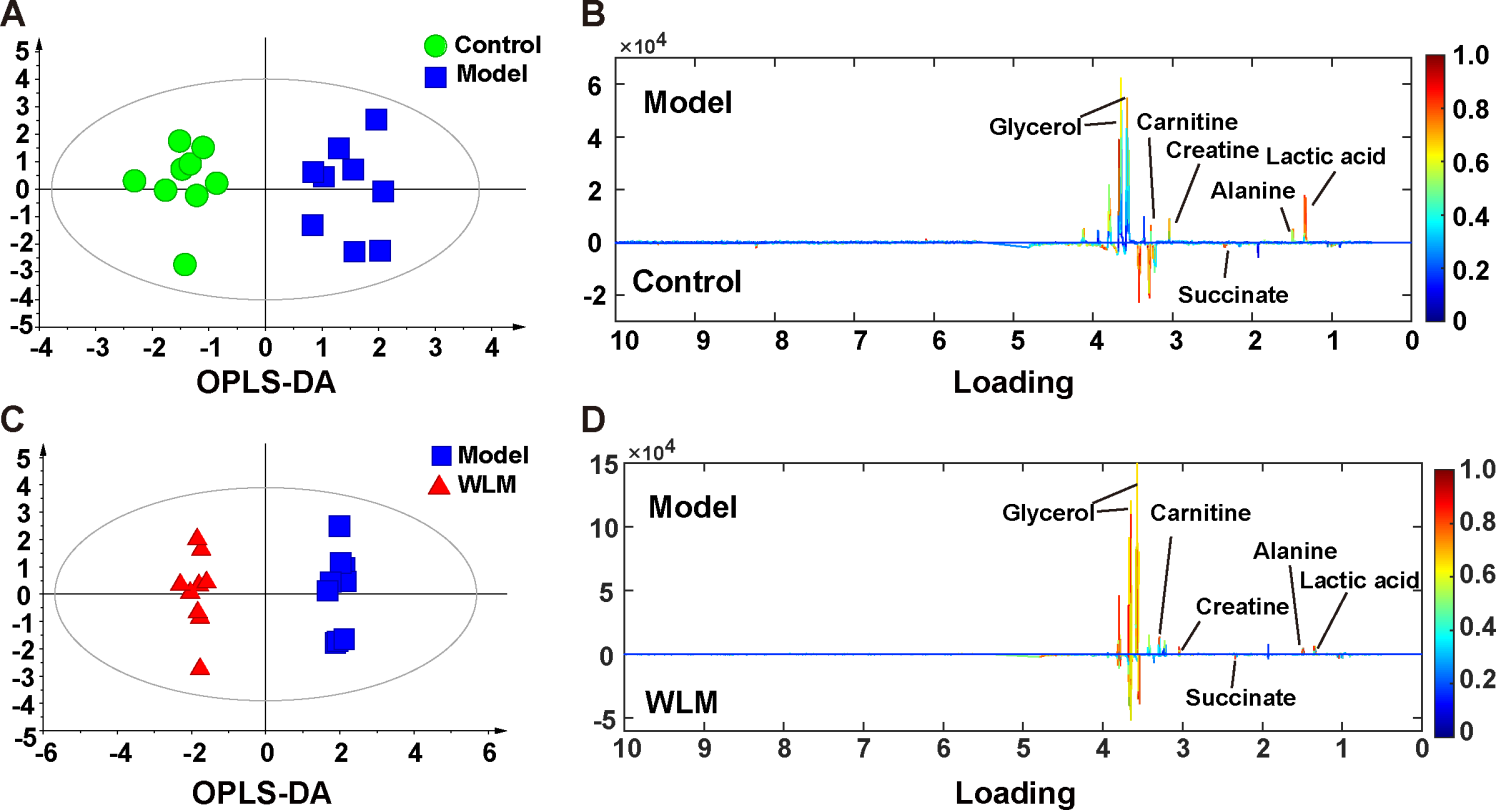
The OPLS-DA score plots. **(A, C)** and the corresponding loading **(B, D)** plots of the ^1^H NMR spectra from the colonic tissue of UC mice; the parameters of OPLS-DA model were as follows: A; model *vs.* control R^2^ = 0.31; Q^2^ = 0.382; C; WLM *vs.* model R^2^ = 0.507; Q^2^ = 0.808.

**Supplementary Figure S4** shows the typical ^1^H NMR spectra of colon tissues from the control, 3% DSS, and WLM groups. Multivariate statistical analysis results suggested that the colon sample of control, 3% DSS, and WLM group exhibited obvious separation in PCA plots (**Figure 3B**). To further study the effects of WLM on the metabolism of UC mice, the supervised pattern visual clustering analysis of the OPLS-DA model was performed on the ^1^H NMR data (**Figures 5A, C**). The permutation analysis of OPLS-DA model was conducted for validation (**Supplementary Figure S4**), indicated that OPLS-DA score plots were not overfitting. Furthermore, the difference in spectral color in the loading plot indicates the correlation between groups; the correlation coefficient r > 0.6 (red) represents a high correlation (**Figure 5**). Compared to the Control group, the DSS mice showed significantly increased levels of carnitine, creatine, alanine, glycerol, and lactate, whereas the level of succinate was obviously decreased (**Figure 5B**). However, the trend was significantly reversed after treatment with WLM extracts (**Figure 5D**). **Table 2** illustrates the significantly changed metabolites.

**Table 2:** Based on ^1^H NMR for differential metabolites of mice colon.

No.	Metabolites	Moieties	*δ*_H_ (multiplicity)	*δ ^13^C*	Dss/Con	WLM/Dss	HMDB
1	Creatine	CH _3_	3.04 (s)	39.3	**↑**	**↓**	HMDB0000064
		CH _2_	3.94 (s)	56.2			
2	Lactic acid	α-CH	4.11 (q)	71.3	**↑**	**↓**	HMDB0000190
		β-CH _3_	1.33 (d)	23.1			
3	Carnitine	N(CH_3_)_3_	3.23 (s)	#	**↑**	**↓**	HMDB0000062
4	Alanine	α-CH	3.76 (q)	53.3	**↑**	**↓**	HMDB0000161
		β-CH _3_	1.46 (d)	19.1			
5	Succinate	CH _2_	2.39 (s)	37.2	**↓**	**↑**	HMDB0000254
6	Glycerol	CH _2_	3.56 (dd)	64.9	**↑**	**↓**	HMDB0000131
		CH _2_	3.64 (dd)	64.9			

Keys: s, singlet; d, doublet; dd, Doublet of doublets; q, quartet, #, not determined. Dss, DSS group; Con, Control group; WLM, WLM high dose group.

#### 3.5.2 Lipidomics analysis of colon sample based on UHPLC-Q/Trap MS

**Figure 6:**
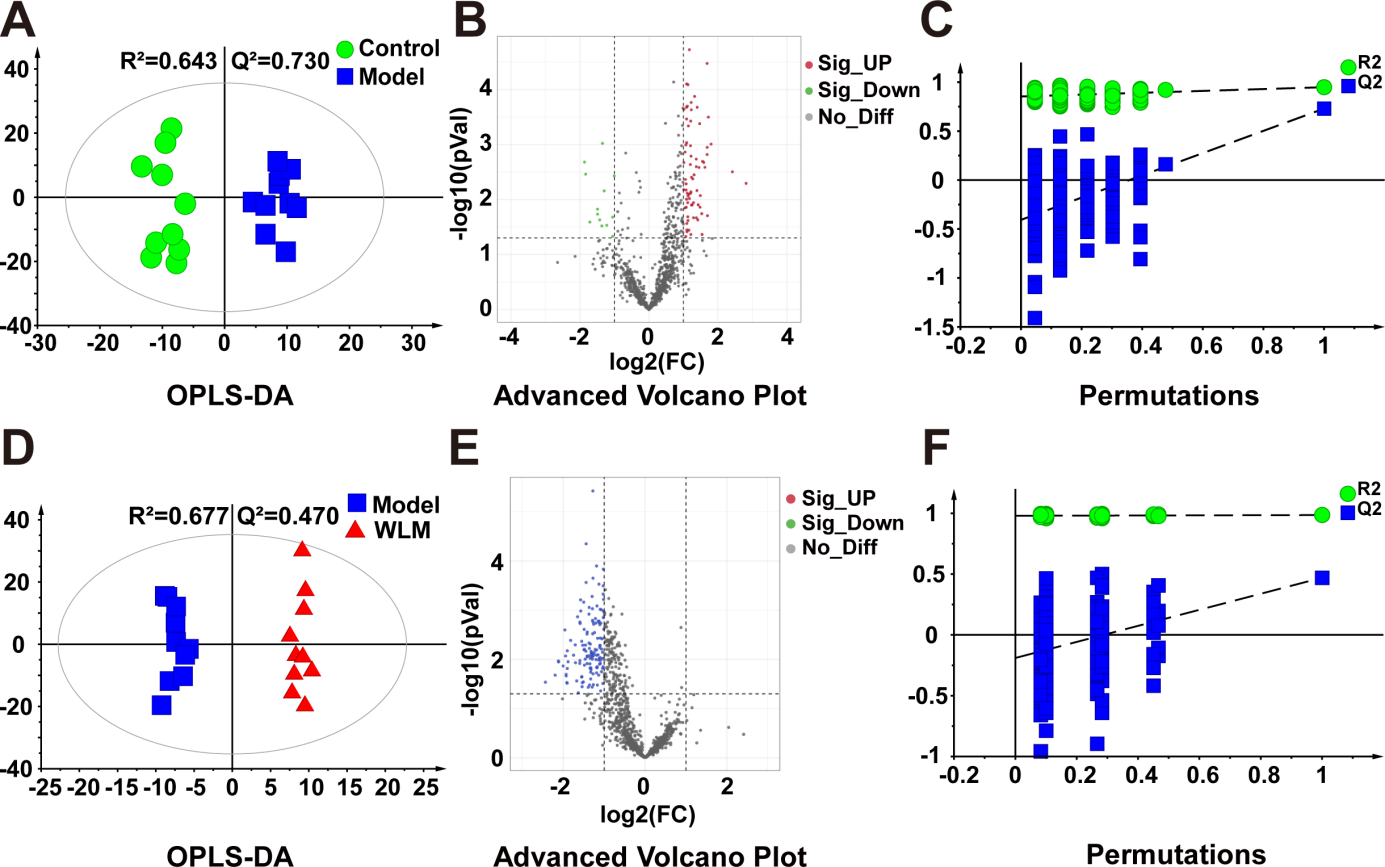
The OPLS-DA score plots. **(A, D)** volcano plot **(B, E)** and permutations analysis **(C, F)** of the UHPLC-Q/Trap MS spectra from colonic tissue of UC mice.

Pseudo-targeted lipidomics employed a multi-reaction monitoring model with 1102 lipid compounds detected. According to the PCA plot of multivariate statistical analysis, the results exhibited significant differences in lipid metabolism among three groups of mice (**Figure 3C**). To screen the differential lipid compounds, the statistical method of OPLS-DA was adopted (**Figure 6**), and the permutation analysis results indicated that the OPLS-DA has no over-fitting. Additionally, the volcano plot screened differential lipid metabolites based on *p* < 0.05 and VIP > 1, and 60 differential lipid compounds were screened (mainly include Phosphatidyl ethanolamine (PE), phosphatidic acid (PA), Phosphatidyl glycerol (PG), and phosphatidylinositol (PI), respectively). **Supplementary Table S5** depicts the differential lipid metabolites. Four kinds of lipid compounds, including PE, PA, PG, and PI, were significantly upregulated compared to the Control group. In contrast, all differential lipid compounds were significantly reversed after treatment with WLM extract.

#### 3.5.3 Metabolic pathway of biomarkers in UC mice

**Figure 7:**
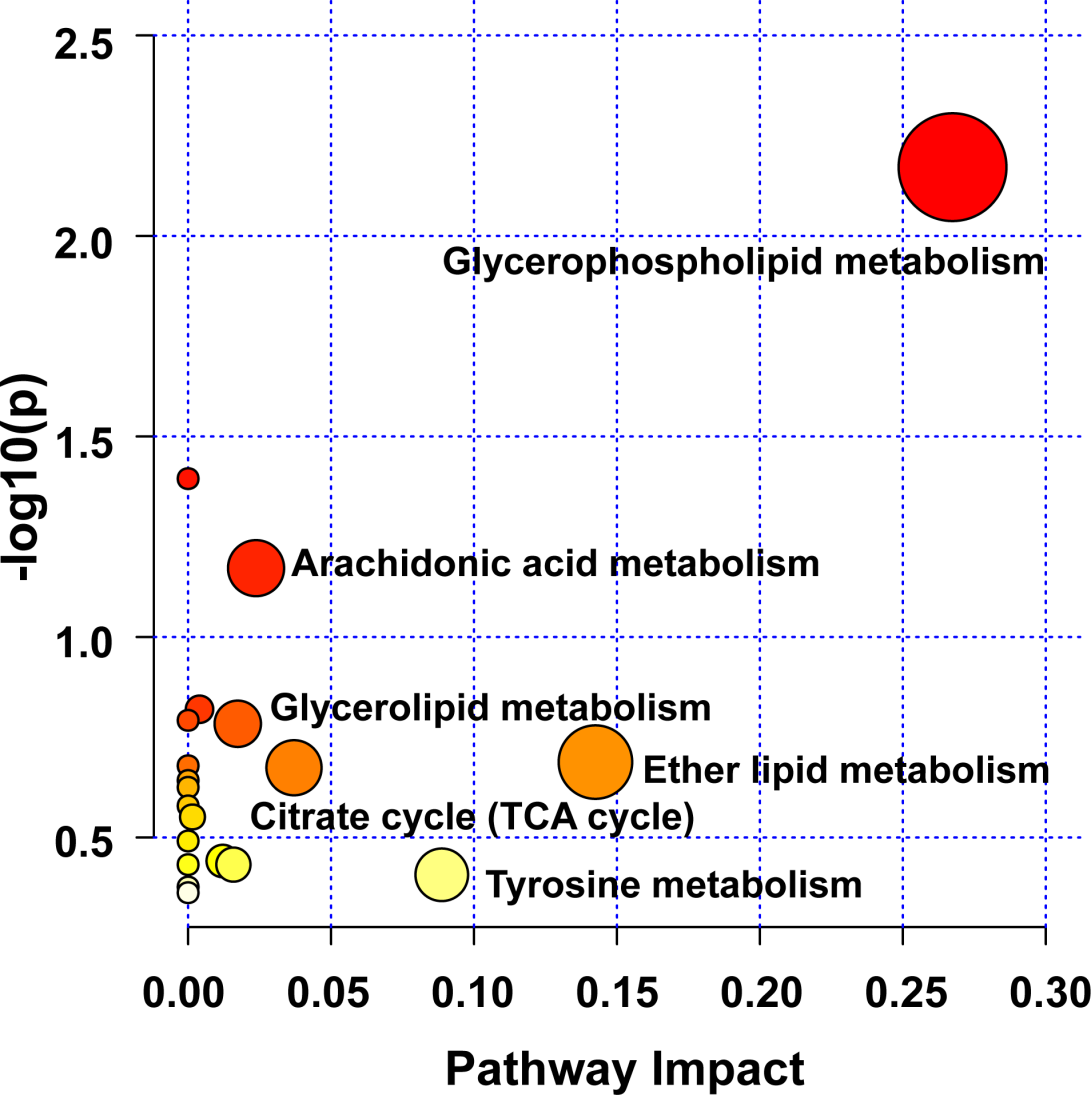
Analysis of UC-related metabolic pathways.

Herein, pathway analysis of all potential biomarkers, including metabolomics and lipidomics screening results, was carried out from the MetaboAnalyst 5.0 platform (https://www.metaboanalyst.ca/). **Figure 7** exhibits the metabolic pathways. It turned out that the efficacy of WLM extract in UC mice was mainly related to six metabolic pathways, including glycerophospholipid, arachidonic acid, glycerolipid, citrate cycle (TCA cycle), ether lipid, and tyrosine metabolisms, which are mainly involved in energy metabolism, inflammatory response, and lipid metabolism.

### 3.6 16S rRNA analysis

**Figure 8:**
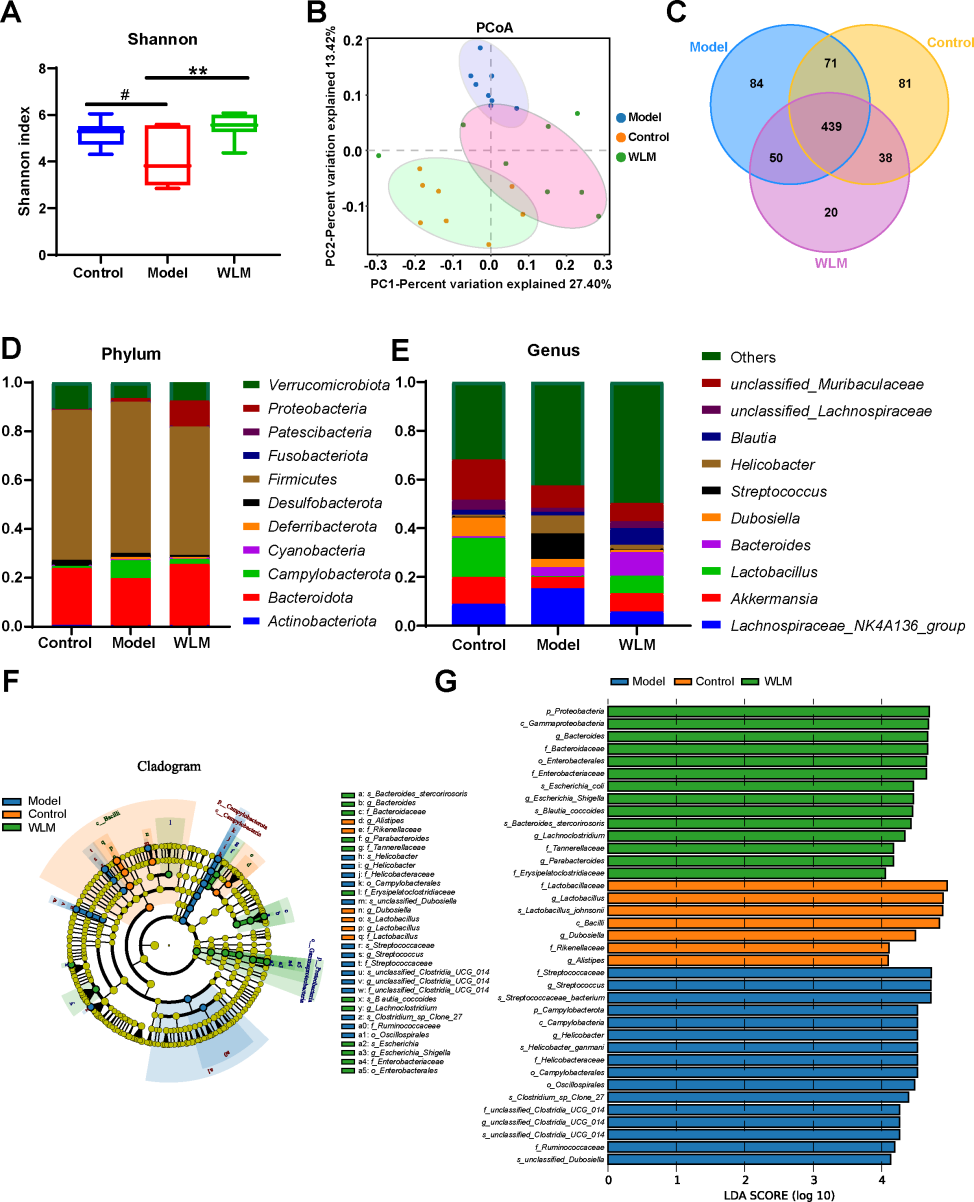
The effect of WLM on gut microbial in UC mice feces. **(A)** Shannon index; **(B)** Principal component analysis (PCA); **(C)** Venn diagram analysis; **(D)** the microbial composition at the phylum level; **(E)** the microbial composition at the genus level; **(F)** LEfSe analysis; and **(G)** Linear discriminant analysis (LDA>4) were used to screen the main microbial characteristic taxa.

We sequenced the 16S rRNA in three groups of fecal samples and analyzed the α and β diversity indices of the gut microbiota in mice. Alpha diversity reflects the richness and diversity of species within a single sample, the Shannon indices was used to measure species diversity, which is influenced by both the richness of species in the sample community and the community evenness. When the species richness is the same, the greater the evenness of the species in the community, the greater the diversity is considered to be. We employed QIIME2 software to conduct a student’s t-test to evaluate the differences in Alpha diversity indices between different treatments. According to Shannon, compared to the DSS and control groups, the gut microbiota diversity was increased in the WLM group (**Figure 8A**). Beta diversity analysis was to compare the similarity of different samples in species diversity. Beta diversity using QIIME software mainly adopts binary jaccard analysis. PCoA analysis revealed significant differences in the composition of gut microbiota among the three groups of mice (P<0.001) (**Figure 8B**). The Control group was significantly separated from the DSS group, indicating significant differences in the gut microbial composition between the two groups of mice. After WLM treatment, the WLM group approached the Control group, indicating that WLM could partially improve the gut microbiota diversity of DSS mice. We also used a Venn diagram to statistically analyze the diversity of microbiota among the three groups as shown in **Figure 8C**. At the phylum levels, Gut bacteria were mainly composed of *Firmicutes*, *Bacteroidota*, *Campylobacterota*, *Verrucomicrobiota1*, of which *Firmicutes*, *Bacteroidota* are the most abundant in relative abundance. (**Figure 8D**). At the genus levels, compared to the DSS group, Gut bacteria were mainly composed of *Lactobacillus*, *Akkermansia*, *Bacteroides*, *Streptococcus*, *Lachnospiraceae* NK4A136 group, the relative abundance of *Streptococcus*, *Helicobacter*, and *Lachnospiraceae* NK4A136 group markedly increased, while the abundance of *Bacteroides*, *Lactobacillus*, and *Akkermansia* significantly decreased (**Figure 8E**). The relative abundance of microbiota was significantly reversed after WLM intervention, suggested that WLM can significantly affect the composition of the gut microbiota in DSS mice.

LEfSe analysis was used to screen for dominant bacteria in the three groups (from phylum to species level) to identify the most significantly different intestinal microbial taxa associated with WLM (**Figure 8F** and **Supplementary Figure S5**). Linear discriminant analysis (LDA) was used to select feature taxonomic groups with significant differences in abundance (**Figure 8G**). At the genus level, compared to the Control group, the relative abundance of *Streptococcus*, *Helicobacter*, and *Lachnospiraceae*_NK4A136_group were increased, while the abundance of *Bacteroides*, *Limosilactobacillus*, *Akkermansia*, and *Enterorhabdus* significantly decreased in Model group (**Supplementary Figure S4**). The relative abundance of microbiota was significantly reversed after WLM intervention, suggesting that WLM can significantly affect the composition of the gut microbiota in DSS mice. These results suggest that the therapeutic effect of WLM on DSS may depend on its regulation of the gut microbiota.

**Figure 9:**
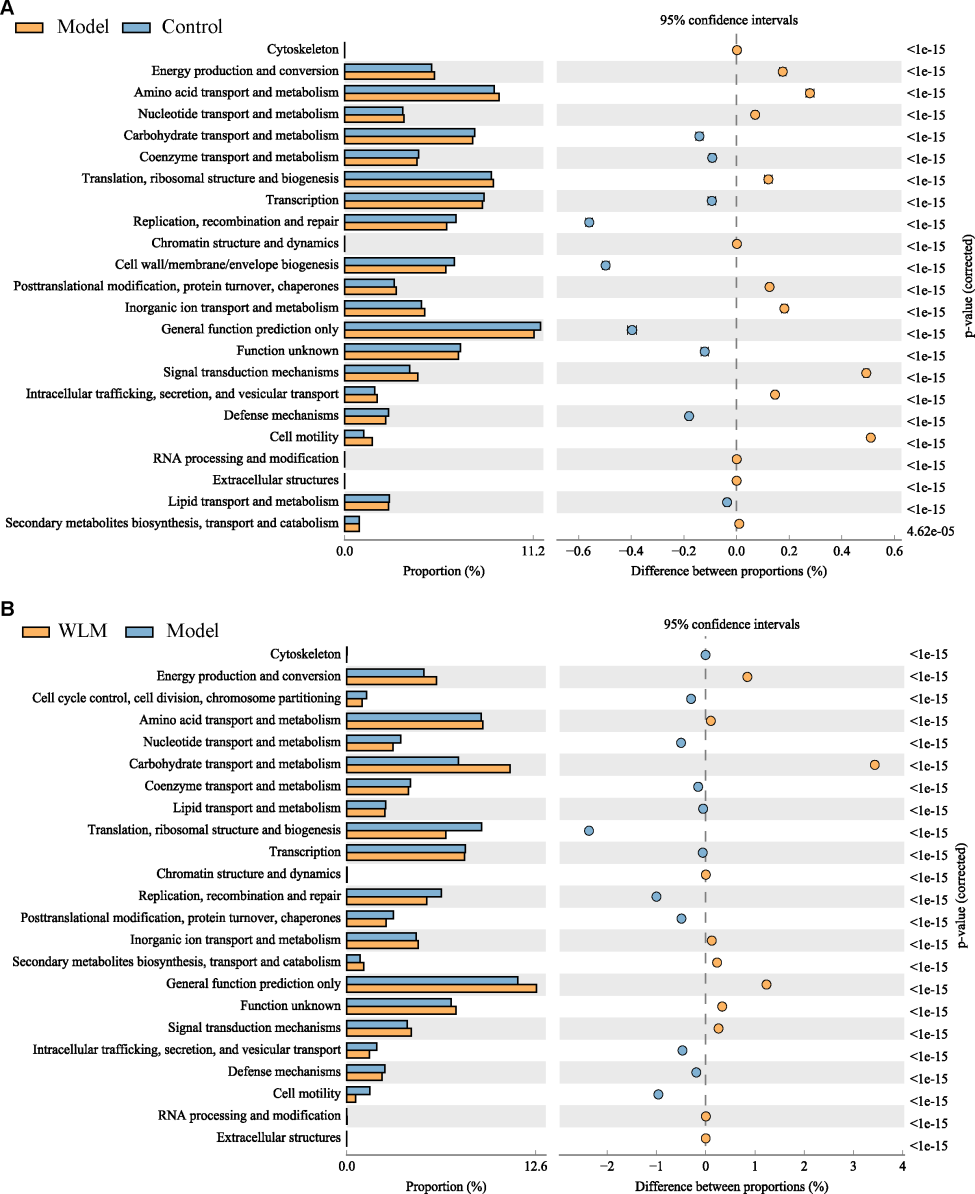
The analysis of gut microbiota-related metabolic pathways. **(A)** Model vs Control; **(B)** WLM vs Model.

To further predict the impact of WLM on the functional profile of the gut microbiota, PICRUST analysis was conducted to assess the functional potentials of the gut microbiota, with subsequent annotation of the predicted outcomes using the KEGG database. As depicted in **Figure 9** and **Supplementary Figure S6**, the results revealed a close association between the microbial community composition and lipid metabolism, energy metabolism, and amino acid metabolism in both the Control and Model groups, suggested that WLM may exert therapeutic effects by modulating alterations in relevant metabolic pathways through the regulation of murine gut microbiota composition, which aligns with our metabolomics findings.

To explore the interaction between differential metabolites, we used spearman correlation analysis to analyze the differential metabolites and inflammatory, and the results were shown in **Supplementary Figure S7**, which suggested that the levels of metabolites related to glycerophospholipid metabolism were negatively correlated with lysophospholipids (**Supplementary Figure S7A**). spearman correlation analysis of LysoPE, LysoPC and inflammatory factors indicated that LysoPE and LysoPC suggested negative correlation with inflammatory factors, the results are shown in **Supplementary Figure S7B**, however, PE, PI, PG, and PA showed positive correlation with inflammatory factors.

We performed Spearman correlation analysis of key metabolites in lipidomics and metabolomics that can be reversed for WLM with gut microbes (**Supplementary Figure S7C**). The results indicated that *Akkermansia*, *Enterorhabdus*, *Prevotellaceae*_NK3B31_group, *Limosllactobacillus* are positively correlated with LysoPC (22:6), LysoPE (22:4/0:0), LysoPC (20:4), whereas *Helicobacter*, *Turicibacter*, *Harryflintia*, *Anaerovorax* showed negative correlation.

### 3.7 Effects of WLM on mRNA expression of inflammatory cytokine

**Figure 10:**
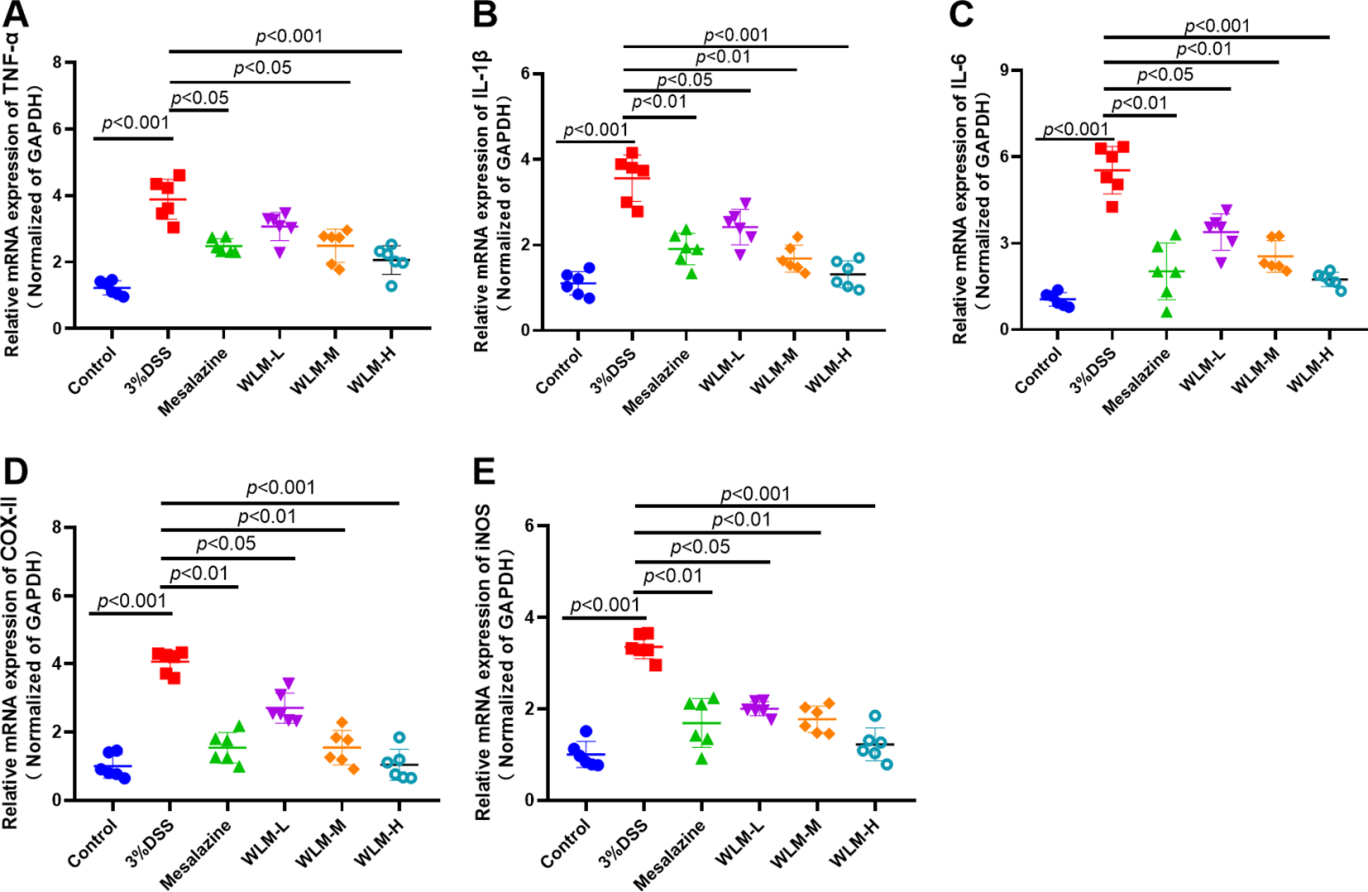
The anti-inflammation effect of WLM on colon tissue in UC mice. TNF-α; **(A)** IL-β; **(B)** IL-6; **(C)** COX-II; **(D)** and iNOS; **(E)** mRNA expression in colon tissue (data represent the mean ± SEM, *n* = 6). Statistical analysis was performed with DSS *vs* control and drug *vs* DSS.

The inflammatory cytokine activity reflects the inflammation level of colonic tissue in UC mice. Herein, the IL-6, TNF-α, and IL-β mRNA activity of the colonic tissue in the DSS mice were activated, compared to the control mice, indicating that inflammation occurred in the colon tissue of UC mice (**Figures 10A–C**). However, the activity levels of inflammatory cytokine were significantly inhibited after one week of treatment with WLM extracts. To further explore the efficacy of WLM in UC mice, the mRNA expressions of pro-inflammatory cytokine COX-II and iNOS in the colon tissue were measured. The results showed that the inflammatory cytokine COX-II and iNOS in DSS mice were increased compared to the control mice (**Figures 10D, E**). Moreover, COX-II and iNOS were significantly eliminated through the WLM extracts treatment compared to the model group. These studies found that WLM could inhibit the mRNA expression levels of pro-inflammatory cytokine and alleviate intestinal inflammation.

## 4 Discussion

Recently, UC has increased worldwide (Du and Ha, 2020). Due to the difficulty of curing and easy relapse of UC and the tendency of cancer, it was listed as one of the modern refractory diseases through the World Health Organization (Teng et al., 2021). The pathogenesis of disease was unclarified clinically; accordingly, there is an urgent need to explore new and effective treatment strategies to control the symptoms better. WLM mainly contains flavonoids and alkaloids, such as apigenin, genistein and berberine, which can effectively relieve the clinical symptoms of UC. Studies have shown that apigenin effectively ameliorated DSS-induced UC via balancing gut microbiome to inhibit inflammation and protect gut barrier (Fu et al., 2022). Berberine hydrochloride has a significant protective effect on ulcerative colitis induced through the DSS (Zhu et al., 2019). This study demonstrated the anti-UC activity and mechanism of action of WLM extracts based on pharmacodynamics, metabolomics, gut microbiota, and molecular biology. The main pathological characteristics of UC mice were analyzed, such as shortened colon length, weight loss, hematochezia, and pathological sections. The above confirmed that WLM had a remission effect on UC to a certain extent. Furthermore, WLM treatment could inhibit the level of inhibiting inflammatory cytokines. Accordingly, we speculated that WLM might be a promising medical candidate for treating UC.

Inflammatory cytokines are closely related to the clinical symptoms of UC. During the acute phase of UC, pro-inflammatory cytokines further stimulate the production of oxidative products, which leads to the disruption of redox balance and intensification of inflammation (Wang et al., 2023). The function of intestinal epithelial barrier can be destroyed through the enhancement of pro-inflammatory cytokine levels. IL-2, IL-1β, IFN-γ, IL-6, TNF-α, and IL-8 is a pro-inflammatory cytokine associated with inflammatory bowel disease, and the pro-inflammatory cytokine response is a critical pathophysiological factor in the acceleration of the occurrence and development of UC (Ungaro et al., 2017). Moreover, sustained inflammatory stimuli can also lead to compromised intestinal barrier function, consequently generating pro-inflammatory cytokines and establishing a vicious cycle (Wang et al., 2023). Our results indicated that the administration of WLM significantly reduced the levels of pro-inflammatory cytokines. These findings suggested that WLM may hold promise as an anti-inflammatory agent for the treatment of inflammatory diseases.

Currently, metabolomics is increasingly used to screen and diagnose biomarkers of UC due to a lack of adequate clinical understanding of the etiology and pathogenesis of UC (Probert et al., 2018). Herein, pseudo-targeted lipidomics and non-targeted metabolomics were used to explain the therapeutic effect of WLM and to excavate its related mechanisms. A total of 21 differential metabolites and 60 lipid metabolites as a biomarker were selected in non-targeted metabolomics and pseudo-targeted lipidomics, respectively, which were closely related to DSS-induced UC. Besides, pathway analysis showed that WLM improved UC by restoring metabolisms such as glycerophospholipid metabolism, arachidonic acid metabolism, glycerolipid metabolism, ether lipid metabolism, tyrosine metabolism, and TCA cycle.

The TCA cycle plays a dominant role in UC. The level of succinic acid in the colon of DSS mice was decreased. However, the content of lactic acid was significantly increased, suggesting that metabolism tended to anaerobic glycolysis, the TCA cycle was disturbed, and the energy supply was damaged (Cai et al., 2021). Generally, carnitine and creatine are involved in the energy supply to cells (Izquierdo-Garcia et al., 2020). Creatine is a major metabolite for storing and utilizing energy. In this work, creatine was significant increase in DSS mice, indicating that the energy supply was disordered in DSS-induced colitis mice. Carnitine is involved in the metabolism of some bacteria, plants, and most mammals and plays an essential role in β-oxidation and lipid metabolism. It is used to transport long-chain fatty acids to mitochondria for oxidation to produce energy, which is a major part of energy metabolism (Zhang et al., 2014). It has been suggested that under the influence of oxidative stress and weight loss, carnitine and creatine were usually increased in the disease development of UC, which may reflect the overall stress state of animals, further indicating the need for ATP and fatty acids to provide energy in the pathological process of UC mice (Connor et al., 2004; Lee et al., 2010).. These differential metabolites play an important role in energy metabolism as potential biomarkers of UC. Interestingly, in our study, this situation was reversed after intervention with WLM, suggesting that WLM had a positive effect on the regulation of stress response and energy supply in UC mice, which was consistent with the results reported in the literature (Kim et al., 2020).

Glycerol has recently been described as a single-molecule systemic biomarker of infection. It has been found that there is a correlation between glycerol signal detected by NMR and genes known to be involved in the pathogenesis of inflammatory bowel disease (Mossotto et al., 2022). Therefore, the increase of glycerol in plasma reflects the metabolic adaptation to intestinal infection and provides energy for survival, which is consistent with our research findings, the content of glycerol in the model group increased significantly, but it decreased significantly after WLM treatment. PE, PA, PG, and PI are the most common phospholipids in the body. There is increasing evidence that phospholipids, as the main component of biofilm, play a significant role in cell signal transduction, cell inflammation, apoptosis, cell migration, and proliferation, which is of great significance to UC (Jiang et al., 2021). As we all know, phosphatidylcholine (PC) and PE play a dominant role in the content of phospholipids. In the inflammation process, the metabolites produced by PC under the action of phospholipase A2 will form large amounts of anti-inflammatory and pro-inflammatory mediators. 15(S)-HETE is an anti-inflammatory lipid mediator that 15-Lipoxygenase catalyzes the production of arachidonic acid and plays a vital role in the metabolism of arachidonic acid. Arachidonic acid is the metabolic product of linolenic acid, and 13-HpODE and 13(S)-HpOTrE is linoleic acid or derivatives of linoleic acid, which are catalyzed by 15-lipoxidase. These potential biomarkers of UC have strong anti-inflammatory activity (Scaioli et al., 2017; Kikut et al., 2022).. Th1e metabolism of arachidonic acid, a significant pathway obtained from the results of metabolomics and lipidomics, plays a key role in UC. Similar to our results, the contents of anti-inflammatory cytokines in UC patients decreased significantly, whereas the anti-inflammatory ability increased significantly after WLM treatment.

DHEA exists in the blood circulation and tissues of animals and humans, which can inhibit inflammation to some extent (De Bus et al., 2021). prostaglandin E2 (PGE2), a metabolite metabolized through arachidonic acid, is related to promoting inflammation. It can enhance tissue damage through LTB4, while DHEA can reduce the production of inflammatory markers such as PGE2 to block tissue damage of inflammation, which is consistent with our results (Vong et al., 2010). Herein, the significantly increased level of DHEA after WLM treatment indicated that DHEA as a biomarker could play a role in treating UC by inhibiting the production of inflammatory mediators and protecting the damage of colonic tissues.

Although the pathogenesis of UC is still unclear, the role of inflammatory cytokines in mediating the formation of colitis is indisputable. Pro-inflammatory factors such as IL-6 and IL-1β are the cytokines that mediate the pathogenesis of colitis (Kermarrec et al., 2019; Nanki et al., 2020; Zhang J. et al., 2022). Moreover, when the expression of i-NOS induced by inflammatory factors is successful, prostaglandins promote the leukocytes to the inflammatory sites and promote the production of NO, causing an inflammatory reaction and COX-II expression to make the body produce inflammation and pain. Moreover, forming inflammation and COX-II expression play a vital role in colon carcinogenesis (Lin et al., 2022). Herein, the cytokines and inflammatory mediators were quantified. The experimental results suggested that the content levels of these pro-inflammatory factors were decreased. Compared to the Model group, the content of pro-inflammatory factors was significantly decreased in the WLM administration group, indicating that WLM might mediate the inflammatory response by decreasing these pro-inflammatory factors and blocking their activities.

It is well known that the gut is an intricate environment with a rich microbiota that maintains the homeostasis of the organism by regulating the dynamic equilibrium of microorganisms, and the imbalance of the intestinal microbiota can result in immune disorders and is involved in the pathogenesis of ulcerative colitis (Cheng et al., 2022; Niu et al., 2023). In the process of UC treatment, the regulation of intestinal flora contributes to the repair of intestinal mucosa, the improvement of inflammatory response, and the enhancement of patient’s immunity, thus alleviating or even curing the disease (Guo et al., 2020). From our results, we can see that WLM promotes the diversity of the intestinal bacterial communities and changes the microbial community composition in UC mice, and in order to understand the differences in microbial compositions among different groups, we analyzed the microbial compositions of the mice at the phylum level and the genus level, respectively. WLM may improve the symptoms of UC mice by reducing the abundance of *Helicobacter* and *Streptococcus* and increasing the abundance of *Limosilactobacillus* and *Akkermansia*.

Studies have shown that *Helicobacter*, one of the pathogens of UC, has a protective effect in the progression of UC, and that an increase in *Helicobacter* abundance can enhance the severity of UC (Shawabkeh, 2022). This is consistent with our results that the abundance of *Helicobacter* was significantly attenuated through WLM treatment, which suggests that WLM can achieve a therapeutic effect on UC by eliminating *Helicobacter*. In addition, studies have shown that *Limosilactobacillus* has antibacterial and immunological as well as intestinal barrier-enhancing properties, and also inhibits inflammation (Liu B. et al., 2022; Yakabe et al., 2022) and prevents the occurrence and progression of colitis by modulating the NF-*κ*B signaling pathway (Liu B. et al., 2022). *Limosilactobacillus* also increases the induction and production of PD-1 T follicular helper cell-dependent IgA, which alters the gut microbiology and prevents the emergence and development of *Limosilactobacillus*. thereby altering gut microbiota and preventing DSS-induced colitis and intestinal dysbiosis (Liu et al., 2021). These results suggest that WLM exerts a therapeutic effect on UC through modulating the abundance of beneficial bacterial genus in the mice intestinal tract and further alleviating DSS-induced colitis.

According to our analyses of microbial and metabolomic profiles, WLM may have reduced the abundance of *Helicobacter* and *Streptococcus* while increasing the abundance of *Limosilactobacillus* and *Akkermansia*, reversing the alterations in phospholipid compounds in DSS-treated mice. Enrichment analysis of metabolic pathways based on differential metabolites suggests that WLM may modulate lipid metabolism, energy metabolism, and amino acid metabolism by regulating the gut microbiota, thereby alleviating UC in mice. In summary, our results indicate that WLM may regulate the production of metabolites by directly influencing the abundance of the gut microbiome.

## 5 Conclusion

This study demonstrated that WLM could significantly alleviate clinical symptoms and colon tissue damage induced UC by DSS in a dose-dependent manner, and the WLM-H group has a better effect on improving intestinal inflammation and restoring metabolic disorder. Non-targeted metabolomic techniques of ^1^H NMR and UHPLC-Orbitrap-MS, pseudo-targeted lipidomics of UHPLC-Q/trap-MS analysis, and 16S rRNA analysis results suggested that modulation of glycerophospholipid, arachidonic acid, and energy metabolism were important factors in treating UC with WLM. Briefly, WLM may be a potential candidate for botanicals in treating UC.

## Data Availability

The datasets presented in this study can be found in online repositories. The names of the repository/repositories and accession number(s) can be found in the article/**Supplementary Material**.
