# Supplementary material for: Non-targeted metabolomics and pseudo-targeted lipidomics combined with gut microbes reveal the protective effects of Causonis japonica (Thunb.) Raf. in ulcerative colitis mice
Source: Front Cell Infect Microbiol. 2024 Oct 15;14:1397735. doi: 10.3389/fcimb.2024.1397735 (PMC11518848; doi:10.3389/fcimb.2024.1397735)
Supplement: Supplementary file 1 [file DataSheet1.pdf]

Electronic Supporting Materials

Non-targeted Metabolomics and Pseudotargeted Lipidomics Based on ^{1}H-NMR and LC-MS Technology to Reveal the Protective Effect of *Cayratia japonica* (Thunb.) Gagnep. on Ulcerative Colitis mice

Hua Huang[1,#], Jie Jiang[1,#], Yihua Fan[2,#], Xufeng Ding[1], Fang Li[1], Chuanxin Liu[3*], and Lijiang Ji[1*]

1 Department of Anorectal Surgery, Changshu Hospital Affiliated to Nanjing University of Chinese Medicine, Changshu, 215500, Jiangsu province, China.

2 Department of Rheumatism and Immunity, Hospital of Chengdu University of Traditional Chinese Medicine, Chengdu, 610075, Sichuan province, China.

3 Endocrine and Metabolic Disease Center, Medical Key Laboratory of Hereditary Rare Diseases of Henan, Luoyang Sub-Center of National Clinical Research Center for Metabolic Diseases, The First Affiliated Hospital, and College of Clinical Medicine of Henan University of Science and Technology, Luoyang, 471003, China.

Hua Huang, Jie Jiang and Yihua Fan are co-first author

*Co-Corresponding author: Lijiang Ji (E-mail: Ji512@163.com) and Chuanxin Liu (E-mail: 15222003775@163.com).

Based on UHPLC-Q/Orbitrap MS/MS analysis from Aqueous Extract of WLM

LC analysis was conducted on an UltiMate 3000 UHPLC system (Thermo-fisher Scientific) equipped with an ACQUITY UPLC HSS T3 column (2.1 × 100 mm, 1.8 μm, Waters, Ireland) at a temperature of 35 °C. The mobile phase consisted of 0.1% formic acid in water (A) and acetonitrile (B), with a gradient elution from 3 to 20% B for 5 min, 20-30% B, 5-11 min; 30-50% B, 11-15 min; 50-65% B, 15-17 min; 65-100 % B, 18-20 min, 100% B. The flow rate was 0.4 mL·min^{-1}.

MS data were acquired on a Thermo-Fisher Scientific Q-Orbitrap mass spectrometer using a HESI source. The HESI parameters were set as follows: capillary temperature and aux gas heater temperature were 320°C and 350°C, respectively; spray voltage was 3.0 kV in the positive mode and 2.8 kV in the negative mode; and sheath gas (N_2) was 40 arbitrary unit (arb) and auxiliary gas (N_2) was 10 arb (N_2, 99.9% purity). The average collision cracking energy (NCE) was ± 35 V while the scanning range was m/z 100−1500. Data acquisition was carried out using Xcalibur 4.2 software (Thermo-fisher Scientific).

Table S1 Evaluation of disease activity index

score	rate of weight loss (%)	degree of loose stools	degree of bleeding
0	0	normal stools	no bleeding
1	1-5	-	-
2	6-10	loose stools (dry)	slight bleeding
3	11-20	-	-
4	＞20	watery diarrhea	gross bleeding

Table S2 Scoring system for histopathology

Score	The severity of inflammation	The lesion depth	Crypt damage	Lesion range
0	none	none	none	none
1	mild	mucosal layer	basal 1/3 damaged	10%-25%
2	general	submucosal layer	basal 2/3 damaged	26%-50%
3	moderate	muscle layer	only surface epithelium intact	51%-75%
4	severe	transmural	entire crypt and epithelium lost	76%-100%

Scoring system for histopathology = The lesion depth + Crypt damage +(The severity of inflammation + Lesion range)/2.

Table S3 Primer sequences used for real-time qPCR amplification.

Gene	Primer	Sequence (5´-3´)
IL-1β	Forward	GACCTTCCAGGATGAGGACA
	Reverse	AGCTCATATGGGTCCGACAG
GAPDH	Forward	AGGTCGGTGTGAACGGATTTG
	Reverse	TGTAGACCATGTAGTTGAGGTCA
TNF-α	Forward	CTTCTCATTCCTGCTCGTGG
	Reverse	TGATCTGAGTGTGAGGGTCTG
IL-6	Forward	AAGCCAGAGTCATTCAGAGC
	Reverse	GTCCTTAGCCACTCCTTCTG
iNOS	Forward	CCCTTCCGAAGTTTCTGGCA
	Reverse	GGCTGTCAGAGCCTCGTGGC
COX-II	Forward	ACTCACTCAGTTTGTTGAGT
	Reverse	TTTGATTAGTACTGTAGGGT

Table S4 Based on UHPLC-Q/Orbitrap MS Identification of Chemical Components from Aqueous Extract of WLM.

No.	Name	Formula	Calculated	Observed	Adduct	RT [min]	MS/MS	Area
1	Chlorogenic acid	$C_{16}H_{18}O_9$	353.08781	353.08606	$[M-H]^-$	0.78	353.08606,263.02505,233.01451,143.01912	7339396
2	Gallic acid	$C_7H_6O_5$	169.01425	169.01341	$[M-H]^-$	2.19	169.01341,125.02327,97.02826,69.03326	175128361
3	Cucurbitarin A	$C_{19}H_{18}O_3$	295.13287	295.13290	$[M+H]^+$	2.60	244.85918,192.10500,145.06084,99.05579	29365401
4	3R-[3R-(3R-Hydroxybutyryloxy)-butyryloxy)buteric acid	$C_{12}H_{20}O_7$	275.11363	275.11368	$[M-H]^-$	2.96	275.11368,257.10312,185.08127,123.08015,89.02309,71.01253	176349978
5	Geniposidic acid	$C_{16}H_{22}O_{10}$	373.11402	373.11340	$[M-H]^-$	3.49	373.11340,355.10306,343.10321,193.05014	13191210
6	Esculin	$C_{15}H_{16}O_9$	339.07216	339.07251	$[M-H]^-$	4.06	339.07251,322.47324,193.05022,177.01855	7178953
7	Caffeic acid*	$C_9H_8O_4$	179.03498	179.03429	$[M-H]^-$	4.17	179.03429,135.04410,109.02827,71.01257	4077542
8	Protocatechuic acid	$C_7H_6O_4$	153.01933	153.01828	$[M-H]^-$	4.46	153.01828,109.02831,95.01249	4216196
9	Esculetin	$C_9H_6O_4$	177.01933	177.01855	$[M-H]^-$	5.08	177.01855,159.04442,147.04416,133.02835,105.03330	192519270
10	Maritimein	$C_{21}H_{20}O_{11}$	449.10784	449.10971	$[M-H]^-$	5.48	449.10971,287.05630,269.04559,125.02325	98773773
11	Apiin	$C_{26}H_{28}O_{14}$	563.14063	563.14140	$[M-H]^-$	6.01	563.1414,383.07745,353.06686,297.07669,117.03333	22573050
12	Kaempferol 3-glucoside-7-glucuronide	$C_{27}H_{28}O_{17}$	625.13990	625.14150	$[M+H]^+$	6.25	625.1415,463.08789,287.05527,153.01839	48288854
13	Suberic acid	$C_8H_{14}O_4$	173.08193	173.08112	$[M-H]^-$	6.48	173.08112,155.07025,128.87689,111.08029,83.04893	9876503
14	Luteolin 7,3'-diglucuronide	$C_{27}H_{26}O_{18}$	639.11919	639.11987	$[M+H]^+$	6.65	639.11987,463.08786,287.05536,153.01831	173769368
15	Indole-3-acetic acid	$C_{10}H_9NO_2$	176.07061	176.07103	$[M+H]^+$	6.76	176.07103,158.06015,130.06537,118.06553	48528441
16	Kaempferol 3-rhamnoside-7-galacturonide	$C_{27}H_{28}O_{16}$	609.14501	609.14660	$[M+H]^+$	6.94	609.1466,463.08911,287.05518,153.01868	49373823
17	Luteolin 5-glucuronide	$C_{21}H_{18}O_{12}$	461.07255	461.07285	$[M-H]^-$	7.17	461.07285,285.04065,133.02840	8740033
18	Luteolin*	$C_{15}H_{10}O_6$	287.05501	287.05524	$[M+H]^+$	7.19	287.05524,269.04211,240.90598,153.01852	297701852
19	Indole-3-carboxaldehyde	C_9H_7NO	144.04549	144.04443	$[M-H]^-$	7.75	144.04443,116.04939,87.92392	10245313
20	Genistein	$C_{21}H_{18}O_{11}$	271.06010	271.06027	$[M+H]^+$	8.38	271.06027,243.06522,153.01849,119.04949	21620957
21	Apigenin	$C_{15}H_{10}O_5$	269.04555	269.04600	$[M-H]^-$	8.40	269.0460,225.0569,183.0450,151.0042	10447619
22	Baicalein 6-glucuronide	$C_{15}H_{10}O_5$	447.09219	447.09265	$[M+H]^+$	8.41	447.09241,271.06027,153.01840	18205832
23	Berberine	$C_{20}H_{17}NO_4$	336.12303	336.12344	$[M+H]^+$	11.25	336.12344,320.09183,292.09702,186.24385,124.67259,84.96040	20084774
24	Palmitoleic Acid	$C_{16}H_{30}O_2$	253.21730	253.21660	$[M-H]^-$	14.31	253.21594,235.20604,217.19449,135.11708,69.07055	26399573

25	Unknown	$C_{16}H_{36}O_2N$	274.27406	274.27426	$[M+H]^+$	16.02	274.27426,256.26364,106.08676,88.07629,70.06591	387460817

* Comparison to a reference compound.

Table S5 Based on UHPLC-Q/trap MS for differential lipid metabolites of mice colon.

No.	lipids	Q1	Q3	Adduct ion	HMDB	Retention Time	DSS *vs* Con			WLM *vs* DSS		
							Log_2FC	$-log_{10}$ (P value)	Trend	Log_2FC	$-log_{10}$ (P value)	Trend
1	PA(16:0/18:1)	673.48	281.25	$[M-H]^-$	HMDB0007858	5.15	0.95	1.61	Sig_Up	-1.13	1.99	Sig_Down
2	PA(16:0/18:2)	671.47	279.23	$[M-H]^-$	HMDB0007860	5.85	1.37	2.74	Sig_Up	-1.28	2.79	Sig_Down
3	PA(18:0/18:2)	699.50	279.23	$[M-H]^-$	HMDB0007861	5.44	1.33	3.88	Sig_Up	-0.84	2.13	Sig_Down
4	PE(14:0/20:3)	712.49	305.25	$[M-H]^-$	HMDB0008836	5.58	1.08	2.17	Sig_Up	-1.58	3.21	Sig_Down
5	PE(16:0/16:0)	690.51	255.23	$[M-H]^-$	HMDB0008923	5.86	0.84	2.05	Sig_Up	-0.97	2.37	Sig_Down
6	PE(16:0/18:1)	716.52	281.25	$[M-H]^-$	HMDB0008926	5.99	0.66	2.21	Sig_Up	-0.78	2.10	Sig_Down
7	PE(16:0/18:3)	712.49	277.22	$[M-H]^-$	HMDB0008929	5.58	0.78	1.79	Sig_Up	-1.10	2.43	Sig_Down
8	PE(16:0/20:1)	744.56	309.28	$[M-H]^-$	HMDB0008933	6.28	0.64	1.60	Sig_Up	-0.94	2.66	Sig_Down
9	PE(16:0/20:3)	740.52	305.25	$[M-H]^-$	HMDB0008935	5.89	0.88	2.09	Sig_Up	-1.29	3.03	Sig_Down
10	PE(16:0/20:5)	736.49	301.22	$[M-H]^-$	HMDB0008939	5.50	0.93	1.99	Sig_Up	-1.04	2.02	Sig_Down
11	PE(18:0/20:2)	770.57	307.26	$[M-H]^-$	HMDB0009000	6.30	0.71	2.23	Sig_Up	-0.71	2.10	Sig_Down
12	PE(18:0/20:3)	768.56	305.25	$[M-H]^-$	HMDB0009001	6.17	0.91	2.61	Sig_Up	-0.89	2.27	Sig_Down
13	PE(18:0/22:5)	792.56	329.25	$[M-H]^-$	HMDB0009010	6.08	0.69	1.93	Sig_Up	-0.85	2.40	Sig_Down
14	PE(18:0/22:6)	790.54	327.23	$[M-H]^-$	HMDB0009012	6.00	0.80	1.95	Sig_Up	-0.88	1.92	Sig_Down
15	PE(18:1/16:1)	714.51	281.25	$[M-H]^-$	HMDB0009023	5.70	0.71	1.67	Sig_Up	-1.15	2.78	Sig_Down
16	PE(18:1/18:1)	742.54	281.25	$[M-H]^-$	HMDB0009025	6.08	0.83	1.92	Sig_Up	-0.96	2.10	Sig_Down
17	PE(18:1/18:2)	740.52	279.23	$[M-H]^-$	HMDB0009027	5.87	1.13	4.09	Sig_Up	-0.88	2.33	Sig_Down
18	PE(18:1/18:3)	738.51	277.22	$[M-H]^-$	HMDB0009028	5.67	0.85	1.90	Sig_Up	-1.27	2.76	Sig_Down
19	PE(18:1/20:1)	770.57	309.28	$[M-H]^-$	HMDB0009032	6.37	0.77	2.43	Sig_Up	-1.27	3.65	Sig_Down
20	PE(18:1/20:2)	768.56	307.26	$[M-H]^-$	HMDB0009033	6.14	0.88	2.44	Sig_Up	-1.29	3.28	Sig_Down
21	PE(18:1/20:3)	766.54	305.25	$[M-H]^-$	HMDB0009034	5.97	0.89	2.37	Sig_Up	-1.44	3.56	Sig_Down
22	PE(18:1/20:4)	764.52	303.23	$[M-H]^-$	HMDB0009036	5.88	0.88	2.10	Sig_Up	-1.44	4.35	Sig_Down
23	PE(18:1/22:4)	792.56	331.26	$[M-H]^-$	HMDB0009042	6.09	0.78	2.38	Sig_Up	-0.88	2.20	Sig_Down

No.	ID	Q1	Q3	Adduct ion	HMDB	Retention Time	DSS *vs* Con			WLM *vs* DSS		
							Log_2FC	$-log_{10}$ (P value)	Trend	Log_2FC	$-log_{10}$ (P value)	Trend
24	PE(18:1/22:5)	790.54	329.25	$[M-H]^-$	HMDB0009043	5.89	1.01	3.64	Sig_Up	-1.19	3.90	Sig_Down
25	PE(18:1/22:6)	788.52	327.23	$[M-H]^-$	HMDB0009045	5.81	0.95	2.98	Sig_Up	-0.75	2.07	Sig_Down
26	PE(18:2/16:1)	712.49	279.23	$[M-H]^-$	HMDB0009089	5.50	1.05	2.86	Sig_Up	-1.26	2.71	Sig_Down
27	PE(18:2/18:2)	738.51	279.23	$[M-H]^-$	HMDB0009093	5.64	1.14	3.34	Sig_Up	-0.97	2.66	Sig_Down
28	PE(18:2/20:1)	768.56	309.28	$[M-H]^-$	HMDB0009098	6.15	1.12	3.70	Sig_Up	-0.90	2.39	Sig_Down
29	PE(18:2/20:2)	766.54	307.26	$[M-H]^-$	HMDB0009099	5.91	0.94	2.83	Sig_Up	-0.96	2.72	Sig_Down
30	PE(18:2/20:3)	764.52	305.25	$[M-H]^-$	HMDB0009100	5.74	1.09	4.10	Sig_Up	-1.02	3.33	Sig_Down
31	PE(18:2/22:4)	790.54	331.26	$[M-H]^-$	HMDB0009108	5.89	0.86	3.17	Sig_Up	-1.02	3.83	Sig_Down
32	PE(18:2/22:5)	788.52	329.25	$[M-H]^-$	HMDB0009109	5.81	1.09	3.46	Sig_Up	-1.02	2.85	Sig_Down
33	PE(18:2/22:6)	786.51	327.23	$[M-H]^-$	HMDB0009111	5.62	1.23	3.77	Sig_Up	-0.99	2.67	Sig_Down
34	PG(16:0/20:3)	771.52	305.25	$[M-H]^-$	HMDB0010578	5.87	0.97	3.35	Sig_Up	-0.99	2.91	Sig_Down
35	PG(18:0/18:0)	777.57	283.26	$[M-H]^-$	HMDB0010602	6.27	0.80	1.85	Sig_Up	-0.76	1.65	Sig_Down
36	PG(18:1/20:3)	797.53	305.25	$[M-H]^-$	HMDB0010623	5.98	0.62	2.41	Sig_Up	-0.84	2.90	Sig_Down
37	PG(18:2/18:3)	767.49	277.22	$[M-H]^-$	HMDB0010651	5.66	0.94	1.82	Sig_Up	-1.28	2.52	Sig_Down
38	PG(18:2/20:3)	795.52	305.25	$[M-H]^-$	HMDB0010653	5.85	0.77	2.48	Sig_Up	-0.97	3.42	Sig_Down
39	PG(18:2/22:5)	819.52	329.25	$[M-H]^-$	HMDB0010657	5.78	0.61	1.31	Sig_Up	-1.21	2.83	Sig_Down
40	PI(16:0/18:1)	835.53	281.25	$[M-H]^-$	HMDB0009782	5.15	1.25	1.90	Sig_Up	-1.28	2.05	Sig_Down
41	PI(16:0/20:2)	861.55	307.26	$[M-H]^-$	HMDB0009786	5.25	1.16	1.44	Sig_Up	-1.33	1.74	Sig_Down
42	PI(16:0/20:3)	859.53	305.25	$[M-H]^-$	HMDB0009787	5.05	1.23	1.90	Sig_Up	-1.20	2.00	Sig_Down
43	PI(16:0/20:4)	857.52	303.23	$[M-H]^-$	HMDB0009789	4.85	1.04	1.85	Sig_Up	-0.74	1.37	Sig_Down
44	PI(16:0/22:4)	885.55	331.26	$[M-H]^-$	HMDB0009793	5.17	1.62	2.58	Sig_Up	-1.30	2.10	Sig_Down
45	PI(18:0/18:0)	865.58	283.26	$[M-H]^-$	HMDB0009808	5.63	0.83	1.38	Sig_Up	-0.78	1.42	Sig_Down
46	PI(18:0/18:2)	861.55	279.23	$[M-H]^-$	HMDB0009809	5.23	1.47	3.38	Sig_Up	-0.78	1.60	Sig_Down
47	PI(18:0/18:3)	859.53	277.22	$[M-H]^-$	HMDB0009810	5.05	1.37	1.67	Sig_Up	-1.48	1.88	Sig_Down

No.	ID	Q1	Q3	Adduct ion	HMDB	Retention Time	DSS *vs* Con			WLM *vs* DSS		
							Log_2FC	$-log_{10}$ (P value)	Trend	Log_2FC	$-log_{10}$ (P value)	Trend
48	PI(18:0/20:3)	887.57	305.25	$[M-H]^-$	HMDB0009813	5.35	1.20	2.64	Sig_Up	-1.10	2.71	Sig_Down
49	PI(18:0/22:4)	913.58	331.26	$[M-H]^-$	HMDB0009817	5.44	1.08	2.28	Sig_Up	-0.93	2.40	Sig_Down
50	PI(18:0/22:5)	911.57	329.25	$[M-H]^-$	HMDB0009819	5.27	1.01	2.18	Sig_Up	-0.82	1.95	Sig_Down
51	PI(18:0/22:6)	909.55	327.23	$[M-H]^-$	HMDB0009821	5.05	1.72	3.50	Sig_Up	-0.99	1.99	Sig_Down
52	PI(18:1/18:1)	861.55	281.25	$[M-H]^-$	HMDB0009824	5.22	1.45	2.58	Sig_Up	-1.15	2.09	Sig_Down
53	PI(18:1/18:2)	859.53	279.23	$[M-H]^-$	HMDB0009826	5.05	1.31	2.45	Sig_Up	-0.80	1.44	Sig_Down
54	PI(18:1/20:3)	885.55	305.25	$[M-H]^-$	HMDB0009830	5.09	1.39	2.16	Sig_Up	-1.36	2.22	Sig_Down
55	PI(18:2/18:2)	857.52	279.23	$[M-H]^-$	HMDB0009850	4.90	1.69	4.47	Sig_Up	-1.13	2.78	Sig_Down
56	PI(18:2/20:1)	887.57	309.28	$[M-H]^-$	HMDB0009852	5.35	1.13	1.62	Sig_Up	-0.94	1.65	Sig_Down
57	PI(18:2/20:2)	885.55	307.26	$[M-H]^-$	HMDB0009853	5.09	1.69	2.95	Sig_Up	-1.22	2.20	Sig_Down
58	PI(18:2/20:3)	883.53	305.25	$[M-H]^-$	HMDB0009854	4.95	1.10	1.56	Sig_Up	-1.32	2.16	Sig_Down
59	PI(20:0/20:3)	915.60	305.25	$[M-H]^-$	HMDB0009868	5.65	1.55	2.69	Sig_Up	-1.24	2.14	Sig_Down
60	PI(20:0/20:4)	913.58	303.23	$[M-H]^-$	HMDB0009869	5.45	0.88	1.40	Sig_Up	-0.81	1.32	Sig_Down

Dss, DSS group; Con, Control group; WLM, WLM high dose group. FC, Fold Change.

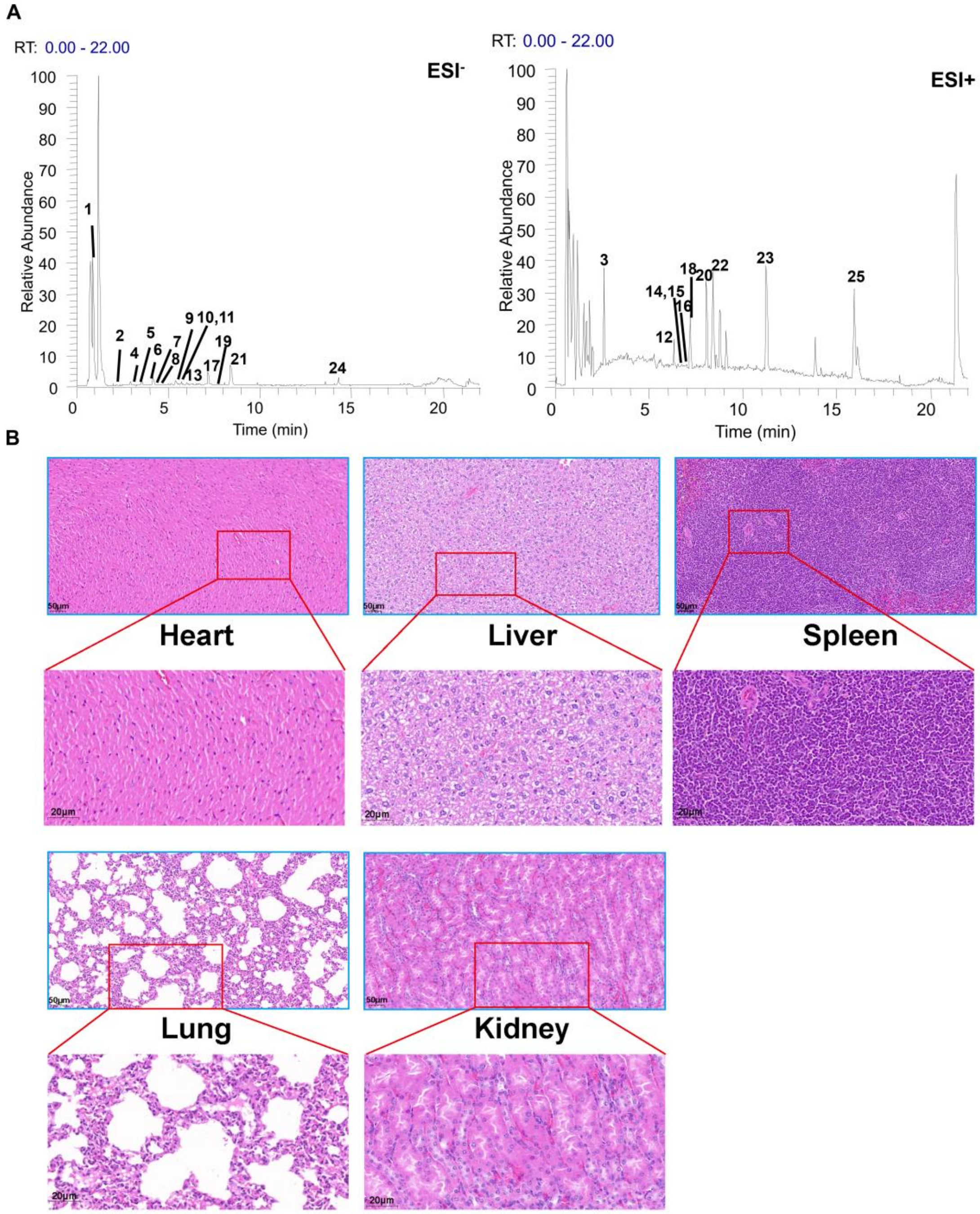

Figure S1 Total ion chromatogram of WLM aqueous extract based on UHPLC-Q/Orbitrap MS (A); HE staining of heart, liver, spleen, lung, and kidney in normal C57 mice after consecutive administration of WLM for 7 days (B).

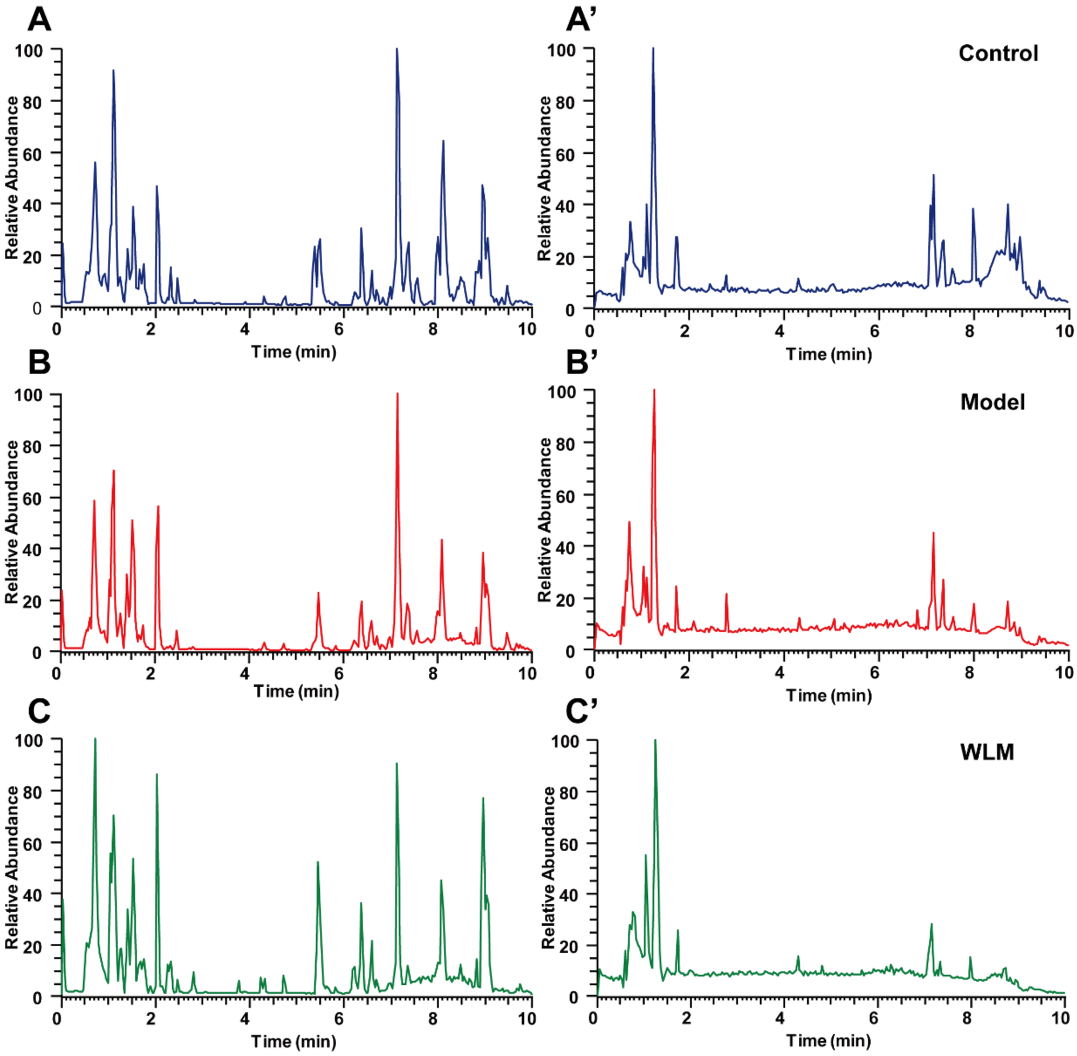

Figure S2 The typical TIC chromatography of mice colon tissue (A, B, and C in the negative mode; A', B', and C' in the positive mode).

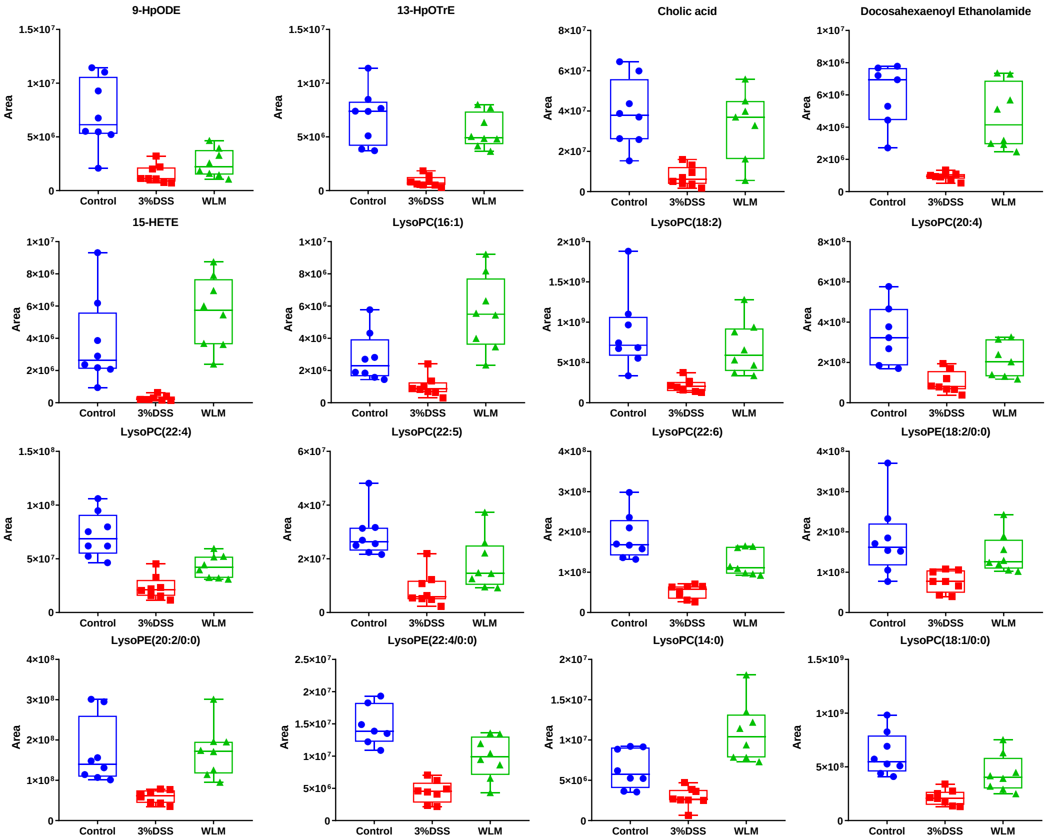

Figure S3 The relative levels of differential metabolites based on UHPLC-Q/Orbitrap MS analysis

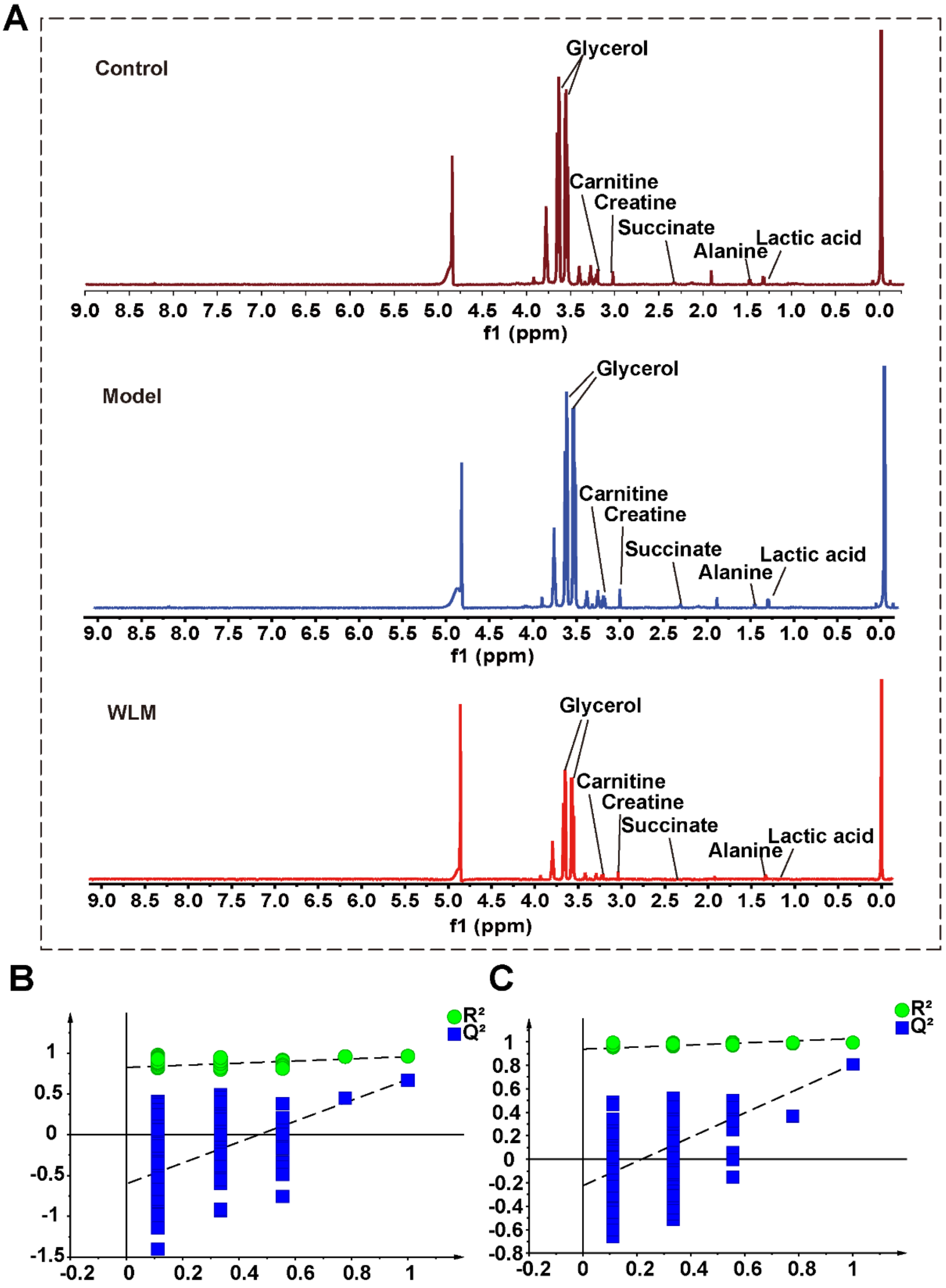

Figure S4 ^{1}H NMR spectra of mice colon tissue from the Control, Model, and WLM group (A). (B) and (C) are permutations analysis for the mice colon tissue based on ^{1}H-NMR analysis, respectively.

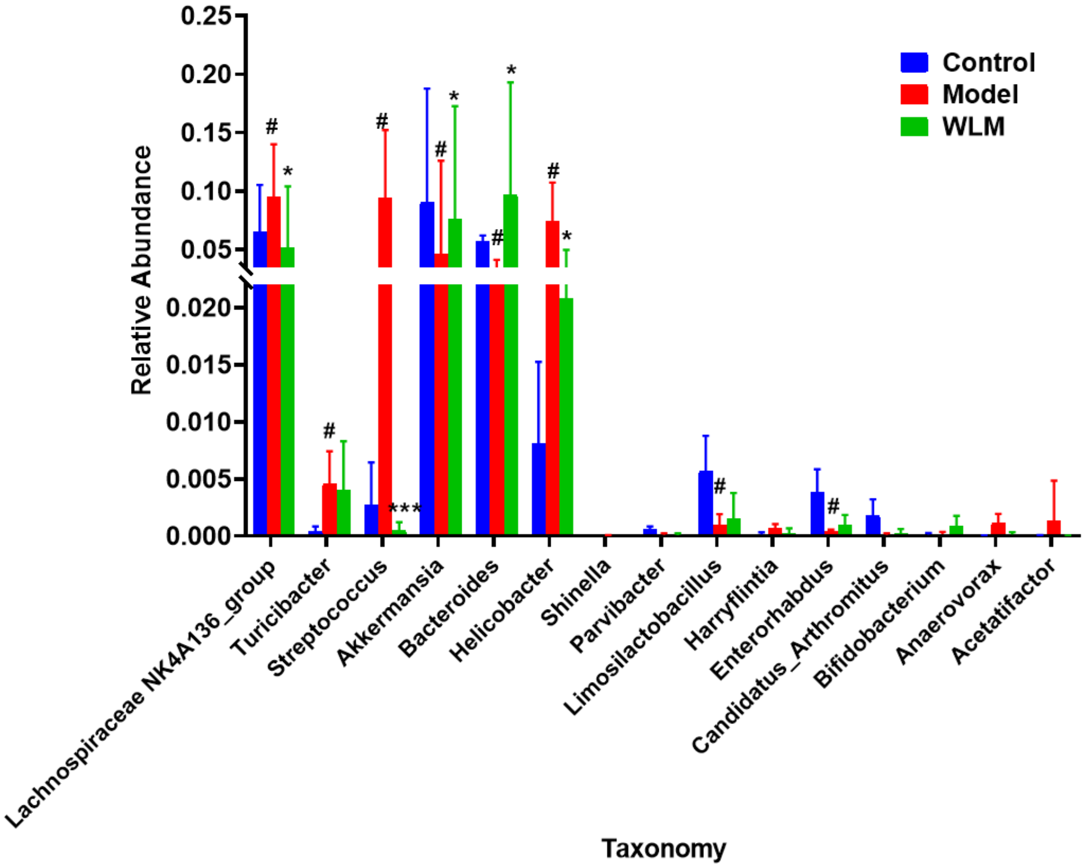

Figure S5 The relative content of differential gut microbes at the genus level.

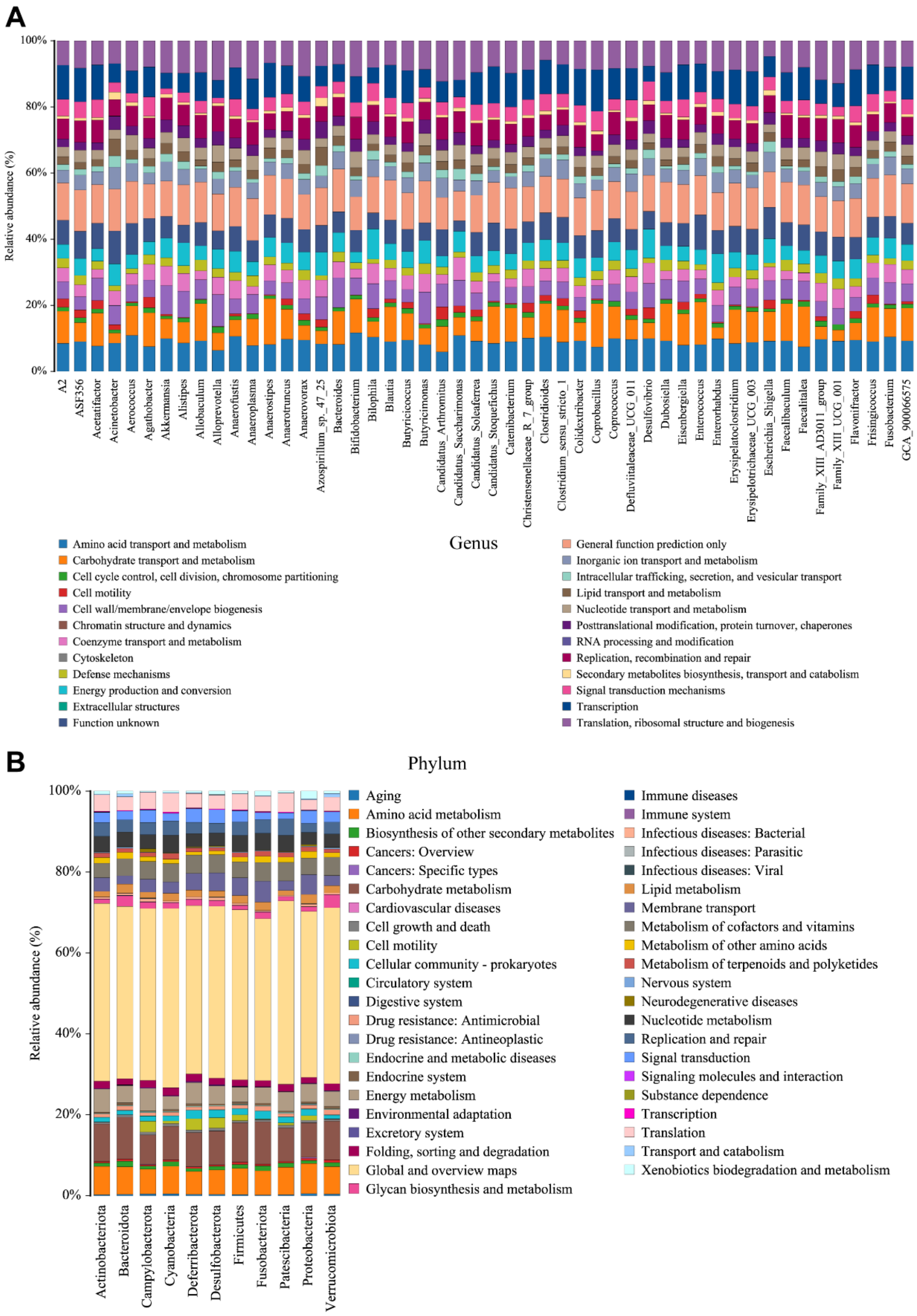

Figure S6 The analysis of metabolic pathways in the gut microbiota at the genus level (A) and phylum level (B).

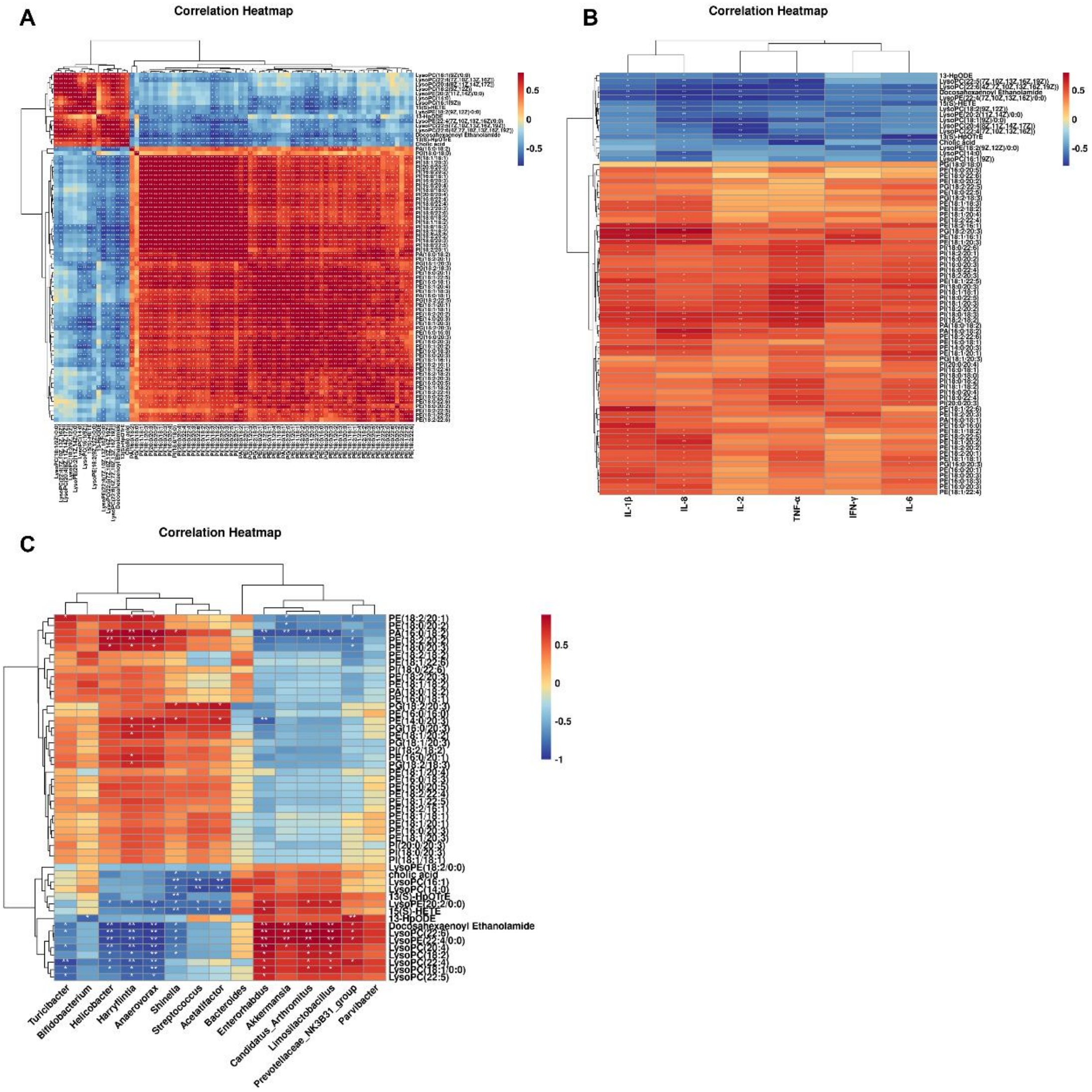

Figure S7 Heat map of spearman correlation matrix between differential metabolites and gut microbiota; (A), spearman correlation matrix heat map between differential metabolites and inflammatory factors (B); spearman correlation matrix heat map between differential metabolites and gut microbiota (C).
